# Loss of *Arabidopsis thaliana* Dynamin-Related Protein 2B Reveals Separation of Innate Immune Signaling Pathways

**DOI:** 10.1371/journal.ppat.1004578

**Published:** 2014-12-18

**Authors:** John M. Smith, Michelle E. Leslie, Samuel J. Robinson, David A. Korasick, Tong Zhang, Steven K. Backues, Peter V. Cornish, Abraham J. Koo, Sebastian Y. Bednarek, Antje Heese

**Affiliations:** 1 Division of Biochemistry, University of Missouri-Columbia, Columbia, Missouri, United States of America; 2 Interdisciplinary Plant Group (IPG), University of Missouri-Columbia, Columbia, Missouri, United States of America; 3 Division of Plant Sciences, University of Missouri-Columbia, Columbia, Missouri, United States of America; 4 Department of Biochemistry, University of Wisconsin-Madison, Madison, Wisconsin, United States of America; University of British Columbia, Canada

## Abstract

Vesicular trafficking has emerged as an important means by which eukaryotes modulate responses to microbial pathogens, likely by contributing to the correct localization and levels of host components necessary for effective immunity. However, considering the complexity of membrane trafficking in plants, relatively few vesicular trafficking components with functions in plant immunity are known. Here we demonstrate that *Arabidopsis thaliana* Dynamin-Related Protein 2B (DRP2B), which has been previously implicated in constitutive clathrin-mediated endocytosis (CME), functions in responses to flg22 (the active peptide derivative of bacterial flagellin) and immunity against flagellated bacteria *Pseudomonas syringae* pv. *tomato* (*Pto*) DC3000. Consistent with a role of DRP2B in Pattern-Triggered Immunity (PTI), *drp2b* null mutant plants also showed increased susceptibility to *Pto* DC3000 *hrcC* ^−^, which lacks a functional Type 3 Secretion System, thus is unable to deliver effectors into host cells to suppress PTI. Importantly, analysis of *drp2b* mutant plants revealed three distinct branches of the flg22-signaling network that differed in their requirement for *RESPIRATORY BURST OXIDASE HOMOLOGUE D (RBOHD)*, the NADPH oxidase responsible for flg22-induced apoplastic reactive oxygen species production. Furthermore, in *drp2b*, normal MAPK signaling and increased immune responses via the *RbohD*/Ca^2+^-branch were not sufficient for promoting robust *PR1* mRNA expression nor immunity against *Pto* DC3000 and *Pto* DC3000 *hrcC^−^*. Based on live-cell imaging studies, flg22-elicited internalization of the plant flagellin-receptor, FLAGELLIN SENSING 2 (FLS2), was found to be partially dependent on DRP2B, but not the closely related protein DRP2A, thus providing genetic evidence for a component, implicated in CME, in ligand-induced endocytosis of FLS2. Reduced trafficking of FLS2 in response to flg22 may contribute in part to the non-canonical combination of immune signaling defects observed in *drp2b*. In conclusion, this study adds DRP2B to the relatively short list of known vesicular trafficking proteins with roles in flg22-signaling and PTI in plants.

## Author Summary

Plants have developed effective mechanisms for protection against pathogens including bacteria, but if a plant is unable to induce defenses, pathogenic bacteria invade and colonize the host, which can lead to reduced yield and nutritional quality of crops. An important aspect of engineering durable crop resistance against bacteria is elucidating and manipulating resistance pathways in the model plant *Arabidopsis thaliana*. The plant receptor FLAGELLIN SENSING 2 (FLS2) recognizes the bacterial protein flagellin to initiate host defense responses contributing to immunity. Here, we identify Dynamin-Related Protein 2B (DRP2B), previously implicated in membrane trafficking in plants, as a novel component of defense responses against flagellin and bacterial *Pseudomonas syringae* strains in *Arabidopsis thaliana*. More specifically, DRP2B functioned in the first line of defense against bacteria, namely in pattern-triggered immunity. We also demonstrated that DRP2B has different roles in three distinct branches of the flg22-signaling network that could be separated by their genetic requirement for the NADPH oxidase *RbohD*. In *drp2b* mutant plants, impaired ligand-induced endocytosis of FLS2 may contribute in part to the non-canonical combination of immune defects. Our findings highlight the importance of a functional vesicular trafficking network for plant immune responses and effective immunity against bacteria.

## Introduction

Eukaryotes have developed highly effective immune mechanisms for protection against microbial pathogens. As the first line of defense, Pattern-Triggered Immunity (PTI) relies on the perception of conserved microbial features called Pathogen- (or Microbe-) Associated Molecular Patterns (PAMPs or MAMPs) by host receptors referred to as Pattern Recognition Receptors (PRRs) [1]–[3]. The bacterial PAMP flagellin is the main proteinaceous component of flagellum filaments essential for mobility of pathogenic bacteria such as *Pseudomonades* to infect hosts [4], [5]. In the model plant *Arabidopsis thaliana*, flagellin or its 22 amino acid active peptide-derivative, flg22, is perceived by the extracellular domain of FLAGELLIN SENSING 2 (FLS2), a receptor kinase localized to the plasma membrane (PM) [6]–[8]. In response to flg22, FLS2 undergoes ligand-induced endocytosis and trafficking through the Trans-Golgi Network/Early Endosomes (TGN/EE) and Multi Vesicular Bodies/Late Endosomes (MVB/LE) for subsequent degradation [9]–[14]. Endocytic degradation of FLS2 results in cellular desensitization to flg22, and subsequent new synthesis of FLS2 leads to flg22-resensitization [13]. Overall, relatively few components functioning in ligand-induced endocytosis of FLS2 have been identified, and potential role(s) of FLS2 endocytosis in flg22-signal initiation and/or attenuation remain mostly undefined.

Flg22-binding to FLS2 also initiates a plethora of flg22-signaling responses that include an increase in cytosolic calcium ([Ca^2+^]_cyt_), the production of apoplastic reactive oxygen species (ROS), activation of mitogen-activated protein kinases (MAPKs), transcriptional changes of defense marker genes, production of the plant defense hormone salicylic acid (SA), as well as callose deposition at cell wall sites [7], [15], [16]. Furthermore, flg22 induces rapid changes in the phosphorylation status of many proteins located at the PM as shown by large-scale phosphoproteomics analyses [17], [18]. Increasing evidence indicates that these immune responses form a signaling network consisting of multiple, parallel signaling branches rather than a single linear pathway. However, few mutants and chemical inhibitors have been identified showing separation of these proposed PAMP-signaling branches [13], [19]–[23]. Open questions regarding the network regulation include what components contribute to signal initiation/attentuation through specific branches of the signaling network and whether one signaling branch influences the other(s).

To gain insight into these questions, we used a candidate-based approach focusing on *Arabidopsis thaliana* Dynamin-Related Protein 2B (DRP2B), a vesicular trafficking protein that is phosphorylated in response to flg22 [17], [18], thus potentially placing DRP2B in the flg22-signaling network. As a member of the superfamily of high molecular weight GTPases with roles in membrane dynamics, DRP2B shares 93% amino acid sequence identity with DRP2A [24], [25]. Consistent with these two closely related DRP2s being functionally redundant in growth and development, *drp2a drp2b* double mutants arrest early in female and male gametophyte development, resulting in lethality [26], [27]. DRP2A and DRP2B are the only *bona fide* (or “classical”) dynamins in *A. thaliana* [25], [26] as they share the same domain structure as mammalian Dynamin 1 and 2. Classical mammalian Dynamins are key components of clathrin-mediated endocytosis (CME) catalyzing the scission and release of clathrin-coated vesicles (CCVs) from the PM [28], [29]. In addition to CME, dynamins contribute to endocytic mechanisms that are independent of clathrin [30], [31]. Consistent with its localization to the PM [17], [18], [32], [33], *A. thaliana* DRP2B and a related rice *Oryza sativa Os*DRP2 (Brittle Culm3; BC3) are implicated in CME based on their co-localization with clathrin-light chain [33], [34] as well as a functional study, in which roots of *Arabidopsis* mutants that express dominant-negative DRP2A/B show defects in the uptake of the endocytic tracer dye, FM4-64 [27]. However, it cannot be excluded that plant DRP2s, like their mammalian counterparts [30], [31], play roles in clathrin-independent endocytosis in plants [35], [36]. In rice, the *bc3* allele disrupts cellulose biosynthesis of the secondary cell wall, likely by affecting trafficking of a cellulose synthase catalytic subunit [34], [37]. However, the roles of DRP2s in other cellular responses, including plant innate immunity, remain undefined.

Here, we identified DRP2B to function in flg22-signaling and innate immunity against *Pseudomonas syringae* pv *tomato* (*Pto*) DC3000 and *Pto hrcC^-^*. We provide evidence that DRP2B differentially contributes to three distinct branches of the flg22-signaling network. Moreover, we separated the individual flg22-signaling branches based on the genetic requirement for *RbohD*, which encodes the NADPH oxidase necessary for PAMP-induced apoplastic ROS production. Live-cell imaging studies indicate that DRP2B, but not DRP2A, is required for robust flg22-induced endocytosis of FLS2, and impaired ligand-induced trafficking defects of FLS2 may contribute in part to the non-canonical combination of phenotypic immune defects observed in *drp2b*.

## Materials and Methods

### Plant materials and growth conditions

*drp2a-1*, *drp2a-3*, *drp2b-2*, *drp2b-5*, *sid2-2* and *rbohD* were previously described [26], [38]–[40]. All mutants are in Col-0 ecotype background. The *drp2b-2 rbohD* double mutant was generated by cross between *drp2b-2* and *rbohD*. *drp2b-2* plants expressing apoaequorin (*drp2b-2/AEQ)* or FLS2-GFP (*drp2b/FLS2-GFP*) were generated by crossing *drp2b-2* with Col-0 plants expressing *AEQ* [41] or *FLS2pro:FLS2-3xMyc-EGFP* [9], respectively. Each double mutant was identified by PCR genotyping using primers listed in S1 Table. Arabidopsis seedlings and plant growth was at 22°C as described [38] in a 8-hour light/16-hour dark cycle photoperiod at 82 µmol m^−2^ s^−1^. Except when noted, fully expanded rosette leaves from five-to-six week old plants were used for all assays. After cutting leaf tissue or transfer of seedlings, all samples were floated on dH_2_O overnight at 22°C in continuous light (unless noted otherwise) to reduce wounding response prior to any assays.

### PAMPs

PAMP peptides were as described [13], made by GenScript (Scotch Plains, NJ) and used at indicated concentrations.

### Apoplastic ROS production

Luminol-based ROS production in leaf tissue was performed as described [38] using indicated PAMP concentrations or using *Pto* DC3000 or *Pto hrcC^−^* [42]. For inhibitor treatments, leaf tissue was pre-treated by floating leaf discs in water-containing 30 µM Wortmannin (Wm; Sigma-Aldrich; St. Louis, MO) for one hour prior to subsequent flg22-elicitation [13]. For ROS assays in six-to-seven-day old plants, cotyledons were cut in half, and the two halves were placed into a 96-well microplate for elicitation. All ROS experiments shown in a same panel were performed in the same 96-well plate at the same time to allow for direct comparison.

### Cytosolic calcium measurements

Calcium assays were done as in [43] with minor changes. Leaf discs (1.1 cm^2^) of five-to-six week old plants or cotyledons of eight-day old seedlings grown on agar plates were cut into halves and placed in individual wells of a 96-well microplate with 150 µL reconstitution buffer [10 µM coelenterazine (Nanolight Technology, Pinetop, AZ), 2 mM MES buffer (pH 5.7), 10 mM CaCl_2_]. Prior to elicitation, the solution was removed and 0.1 µM flg22 of ddH_2_O was added to each well. Luminescence was acquired using a Glomax 96 microplate Luminometer (Promega) scanning each row in five-second intervals. Calcium concentration was calculated as described [41].

### Bacterial pathogen assays

*Pto* DC3000*lux* [44] and *Pto* DC3000 *hrcC* [45] were used in leaf tissue at a concentration of OD_600_ = 0.0005 and OD_600_ = 0.02, respectively, as previously described [20]. Bacterial infection of two-week old plants were performed as in [46] with the exception that plants were grown on MS agar plates under a 8-hour light/16-hour dark cycle photoperiod at 114 µmol m^−2^ s^−1^. Prior to infection, four two-week-old plants were transferred to a microtiter plate containing 2 mL of dH_2_O, incubated for 15–20 h prior to infection with *Pto* DC3000*lux* (2×10^7^ cfu/mL). For quantification of bacterial growth, seedlings were rinsed in dH_2_O and ground in 500 µL dH_2_0 prior to serial dilution plating.

### Quantitative Real-Time PCR (qRT-PCR) analysis

Three leaves of five-to-six week old plants were syringe infiltrated with flg22 or bacterial solution at indicated concentrations or optical densities (OD), respectively, allowed to dry and then placed at 22°C. Tissue was flash-frozen in liquid nitrogen at indicated times. Total RNA was isolated from collected tissue using Trizol Reagent (Sigma) according to the manufacturer's protocol and processed for qRT-PCR as described previously [46], [47] using gene-specific primers (S1 Table) and *At2g28390* as a reference gene.

### Immunoblot analysis

For immunoblot analyses of elicited samples, three 1.5 cm^2^ leaf discs (each cut into five strips to maximize exposure of tissue to the elicitation solution) or three seedlings were elicited with indicated flg22-concentrations for specified times and flash frozen in liquid nitrogen following PAMP removal. Sample preparation and immunoblot analysis of total proteins was done as described [38] using antibodies at the dilutions: αDRP2, 1∶2000; αMPK6, 1∶5000, αCalnexin, 1∶3000; αFLS2, 1∶2500; αP-p44/42 MAPK, 1∶3000 (#4370; Cell Signaling Tech, Danvers, MA); αGFP, 1∶2000 (#A-11122; Life Technologies, Grand Island, NY).

### Callose deposition and quantification

Leaves were syringe-infiltrated with indicated concentrations of active or inactive flg22. 24 hours post-infiltration, leaf discs (1.5 cm^2^) were processed using aniline-blue staining as done previously [20], [38]. Callose deposits were visualized by ultraviolent epifluorescence using a Leica M205 FA microscope (Leica Microsystems Inc.; Buffalo Grove, IL, USA) and quantified from digital images as a percentage of each leaf disc covered with bright pixels above a set pixel intensity threshold using Metamorph software (Molecular Devices, Sunnyvale, CA) [48].

### Salicylic Acid (SA) measurement and treatment

Plants were grown and treated as done for pathogen infection assay of two-week old plants. SA content in plant tissue was measured using ultra performance liquid chromatography electrospray ionization tandem mass spectrometry (UPLC-ESI-MS/MS) method as described previously [49], [50] with minor modifications. Briefly, around 50 mg of frozen tissue was ground to a fine powder using a tissue homogenizer (TissueLyser II, Qiagen, Venlo, Netherlands) in a tube containing metal beads. 150 µL of extraction buffer (70% methanol/water (v/v) with 0.5% acetic acid) containing known amount of an internal standard, deuterium labeled -SA (d4-SA), was added to the ground tissue and incubated for 30 min at 4°C while mixing. Following a brief centrifugation, the supernatant was transferred to a microcentrifuge tube and centrifuged at 18,000 *g* for 30 min at 4°C. Five µL of the resulting supernatant was separated on a UPLC BEH C18 column (1.7 µm, 2.1×50 mm; Waters, Milford, MA, USA) attached to an Acquity UPLC H-Class system (Waters) using a 3-min gradient program consisting of 0.15% aqueous formic acid and methanol as mobile phase (0.4 mL/min flow rate at 40°C). Characteristic MS transitions of m/z 137>93 and 141>97 for SA and d4-SA, respectively, were detected using a Xevo TQ-S tandem quadrupole mass spectrometer (Waters) operated at ESI negative ion mode. Peak area was integrated using MassLynx 4.1 software (Waters), and the absolute amount of SA was determined by comparing the relative peak area of SA and d4-SA in the plant extracts to the dose-response curve generated using pure SA and d4-SA standards.

For exogenous application of SA, four two-week-old plants were transferred from MS agar plates to a single well of a 24-well microtiter plate containing 2 mL of dH_2_O and incubated at 22°C for 15–20 hours. At the time of treatment, dH_2_O was removed from individual wells of the microtiter plate and replaced with 50 µM SA (Sigma) solution or dH_2_O (mock-treatment). The microtiter plate was returned to the growth chamber, and tissue was flash frozen in liquid nitrogen 24 hours post treatment for subsequent qRT-PCR analyses of *PR1* mRNA levels.

### Spinning disc confocal microscopy

Six-day old Col-0 *WT FLS2-GFP* and *drp2b-2 FLS2-GFP* seedlings were transferred to 0.6 mL dH_2_O in a 48-well tissue culture plate (two to three seedlings/well) and incubated at 22°C with continuous light for 16–20 hours. Untreated (0 min flg22) seedlings were mounted in water and immediately imaged. For imaging flg22-induced endocytosis of FLS2, seedlings were submerged in 1 µM flg22 for 30 or 45 minutes and then immediately mounted for imaging over a 10-min period (i.e. 35–45 or 50–60 min, respectively). FLS2-GFP was imaged in the epidermal pavement cell layer of the adaxial (top) cotyledon surface. For each experiment, at least four seedlings were imaged per genotype/treatment, with four to ten fields of view per seedling.

Live-cell imaging experiments were carried out using a custom Olympus IX-71 inverted microscope (Center Valley, PA) equipped with a Yokogawa CSU-X1 5000 rpm spinning disc unit (Tokyo, Japan), Andor iXon Ultra 897 High Speed EMCCD camera (Belfast, United Kingdom), PZ-2000 XYZ series automated stage with Piezo Z-axis top plate (Applied Scientific Instrumentation; Eugene, OR), and 60x-silicon oil objective (Olympus UPlanSApo 60x/1.30 Sil). GFP was excited with a Spectra Physics 488-nm diode laser (Santa Clara, CA), and fluorescence was collected through a series of Semrock Brightline 488-nm single-edge dichroic beamsplitter and 500-550-nm bandpass filters (Rochester, NY). Camera exposure time was set to 150 msec. For each image series, 66 consecutive images at a z-step interval of 0.31 µm (20 µm total depth) were captured using Andor iQ2 software (Belfast, United Kingdom).

### Quantitative analysis of FLS2-GFP endocytosis and PM intensity

Z-stack image series were processed and analyzed using Fiji software [51]. Each FLS2-GFP image series was displayed as a maximum-intensity projection (MIP) with brightness and contrast adjusted uniformly for all MIPs. Stomata were removed from the MIP using the *Freehand Selection* tool, and the total pavement cell surface area (µm^2^) was measured. FLS2-GFP-containing puncta (endosomes) were detected using the Advanced Weka Segmentation plug-in for Fiji, which uses Weka machine learning capabilities [52] for trainable selection of image features. In brief, a prototype MIP image with clearly defined FLS2-GFP puncta was used to generate a "*Vesicle Classifier*" that was subsequently applied to all other MIPs. Puncta were counted in the resulting binary images using the *Analzye Particles* function in Fiji with the following parameters: particle *Size* = 0.25–2.5 µm^2^ and *Circularity* = 0.25–1.00. Every MIP image was visually inspected after puncta detection for accuracy, and a manual adjustment of the puncta count was made if necessary. Puncta density was calculated for each MIP image as the number of FLS2-GFP puncta per 1000 µm^2^.

For measuring levels of FLS2-GFP at the PM (FLS2-GFP PM intensity), a single optical section of the epidermal pavement cells with juxtaposed PMs was selected from each SDCM Z-series, or captured independently using the same imaging and experimental parameters described. Using Fiji, bright PM regions were highlighted with the *Oval Selection* tool and subsequently analyzed for mean pixel intensity. The FLS2-GFP PM intensity for each image was calculated as the average value of the pixel intensity measurements from four selected PM regions. For each genotype and treatment, FLS2-GFP PM intensities are reported relative to the FLS2-GFP PM intensity of un-elicited Col-0 *FLS2-GFP*.

### Statistical analysis

Unless stated otherwise, each experiment was done at least three independent times with similar results. Statistical significances based on unpaired Two-tailed student's t-test were determined with Graph Pad Prism4 software (La Jolla, CA).

### Accession numbers

*FLS2 (At5g46330), DRP2B (At1g59610), DRP2A (At1g10290), RBOHD (At5g47910), PR1 (At2g14610).*

## Results

### *DRP2B* is a negative regulator of *RbohD*-dependent ROS production in response to flg22

**Figure 1:**
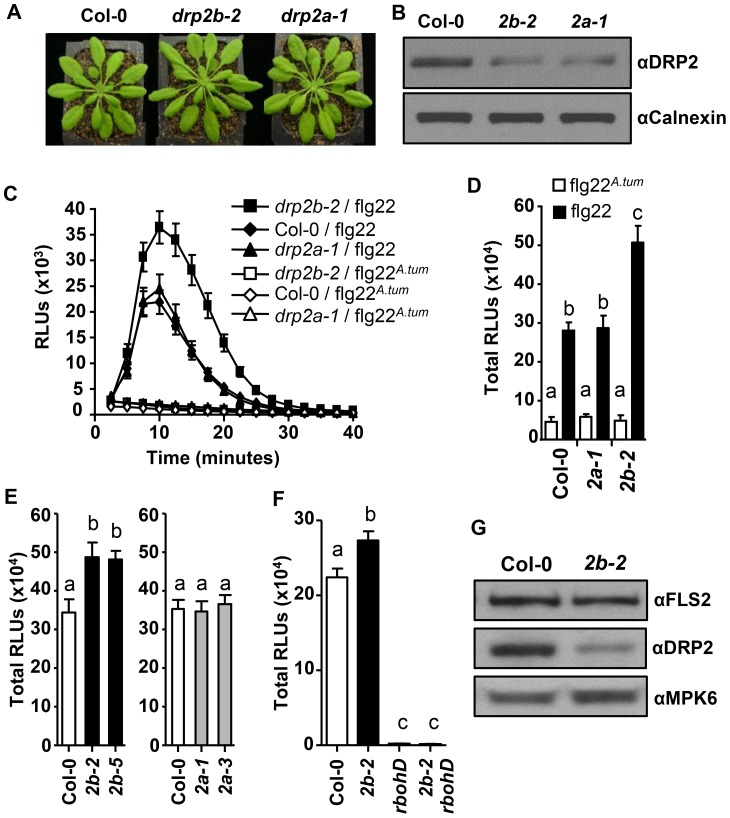
*DRP2B* is a negative regulator of RbohD-dependent ROS Production in response to flg22. (A) No gross growth defects were observed in five-to-six week-old *drp2b-2* (*2b-2*) or *drp2a-1* (*2a-1*) plants relative to the wildtype (Col-0). (B) Compared to Col-0, *drp2b-2* (*2b-2*) and *drp2a-1* (*2a-1*) single mutants exhibited reduced DRP2 protein levels as shown in immunoblot analyses of total protein extracts from un-elicited leaf tissue. αDRP2 antibody showed DRP2 protein levels, and αCalnexin was the loading control. (C) In time-course experiments, ROS production was elevated in *drp2b-2* compared to Col-0 and *drp2a-1* in response to 0.1 µM of active flg22 (filled shapes; n = 24/genotype) but not inactive flg22*^A.tum^* (open shapes; n = 8/genotype). (D) Compared to Col-0 and *drp2a-1* (*2a-1*), total ROS production was significantly increased in *drp2b-2* (*2b-2*) after elicitation with 0.1 µM of active flg22 (P<0.0003) but not in response to inactive flg22*^A.tum^* (P>0.5). Data were based on time-course experiment shown in Figure (C). (E) Independent *drp2b* (black bars), but not *drp2a* (gray bars), null mutant alleles displayed increased flg22-dependent ROS production compared to Col-0 (white bar) (P<0.007) (n = 24/genotype). (F) Similar to *rbohD* single mutants, *drp2b-2 rbohD* double mutants (*2b-2 rbohD*) did not produce any ROS in response to 0.1 µM active flg22 (P>0.125). (n = 24/genotype and treatment). (G) Steady-state protein levels of FLS2 were similar between Col-0 and *drp2b-2* (*2b-2*) as shown by immunoblot analyses of total proteins extracts from un-elicited leaf tissue using αFLS2 and αDRP2 antibodies. αMPK6 served as a loading control. All experiments were done in 5-week old leaf tissue and repeated more than three independent times with similar results. Values are mean ± SE. Different letters indicate significant differences while the same letter indicates no significant differences between samples based on Two-tailed student's t-test. Relative Light Units, RLU.

As previous work has shown that *drp2a drp2b* double null mutants are gametophytic lethal [26], [27], all experiments conducted in this study were performed using our previously published *drp2a* and *drp2b* single null mutants [26]. Overall, these single mutants do not show any gross morphological defects (Fig. 1A) [26], [27]. Using an affinity purified polyclonal peptide antibody (αDRP2) that detects both DRP2A and DRP2B proteins due to their high amino acid sequence identity [26], we confirmed by immunoblot analysis that five-to-six week old leaves of *drp2a-1* (SALK_071036) or *drp2b-2* (SALK_134887) single mutants accumulated significantly reduced levels of DRP2 proteins (Fig. 1B). These results are consistent with reduced DRP2 protein levels seen in *drp2* single mutants at the seedling stage [26]. The residual levels of protein detected by the DRP2 antibody in the *drp2b-2* mutant likely represented DRP2A, and DRP2B in *drp2a-1*.

Analysis of PAMP-elicited ROS has proven to be a valuable tool in identifying and characterizing novel components involved in PAMP-signaling [18], [20], [38], [53], [54]. Therefore, we monitored ROS production in response to flg22 in *drp2* single mutants. In *drp2b-2* but not *drp2a-1* null mutant leaves, flg22-induced ROS production was significantly increased relative to Col-0 when ROS production was plotted over time (Fig. 1C), at its peak at 10–12 minutes post-elicitation (S1A Fig.) and as total ROS produced over 40 minutes (Fig. 1D). In contrast, no significant difference was observed between flg22-elicited ROS in *drp2a-1* and Col-0 (Fig. 1C-D; S1A Fig.). In control experiments, little to no ROS was detected in response to inactive flg22 derived from *Agrobacterium tumefaciens* (flg22*^A.tum^*) [55] in either *drp2a-1, drp2b-2* or Col-0 (Fig. 1C). Similar results were observed using the independent *DRP2* null alleles, *drp2b-5* (SALK_041330) and *drp2a-3* (SALK_011319) [26] (Fig. 1E; total RLUs) confirming that the increased ROS production in *drp2b* mutant lines was specific to loss of *DRP2B*. In *drp2b-2*, ROS production was also significantly increased after elicitation with elf26, an unrelated bacterial PAMP detected by the PRR Elongation Factor-Tu receptor (EFR) [1]–[3] (S1B Fig.), indicating that DRP2B's function is not restricted to flg22. Taken together, our results show that *DRP2B* acts as a negative regulator of flg22-induced ROS production, whereas *DRP2A* has no apparent role in this response.

Next, we investigated whether the increase in amplitude of flg22-induced ROS in *drp2b-2* was dependent on RbohD, the plasma membrane-localized NADPH oxidase responsible for rapid apoplastic ROS production after elicitation with PAMPs [18], [39], [56]. In the homozygous *drp2b-2 rbohD* double mutant, flg22-elicited ROS production was highly reduced and statistically similar to ROS in the *rbohD* single mutant (Fig. 1F). These results indicate that in *drp2b-*2, the increase in flg22-induced ROS production is completely *RbohD*-dependent.

The increased ROS production in *drp2b-2* was unlikely due to increased expression of *RbohD* and *FLS2* because in quantitative reverse transcription polymerase chain reaction (qRT-PCR) experiments, no significant difference in steady-state *RbohD* or *FLS2* mRNA levels was observed between *drp2b-2* and Col-0 (S1C-D Fig.). Furthermore, FLS2 protein levels were found by immunoblot analysis of total protein extracts from *drp2b-2* and Col-0 lines to be similar (Fig. 1G), indicating that changes in the steady-state levels of the FLS2 receptor are not likely to account for the observed increase in flg22-dependent ROS production in *drp2b-2*.

### *DRP2B* negatively regulates flg22-induced Ca^2+^-dependent responses but has no apparent role in MAPK-dependent responses

**Figure 2:**
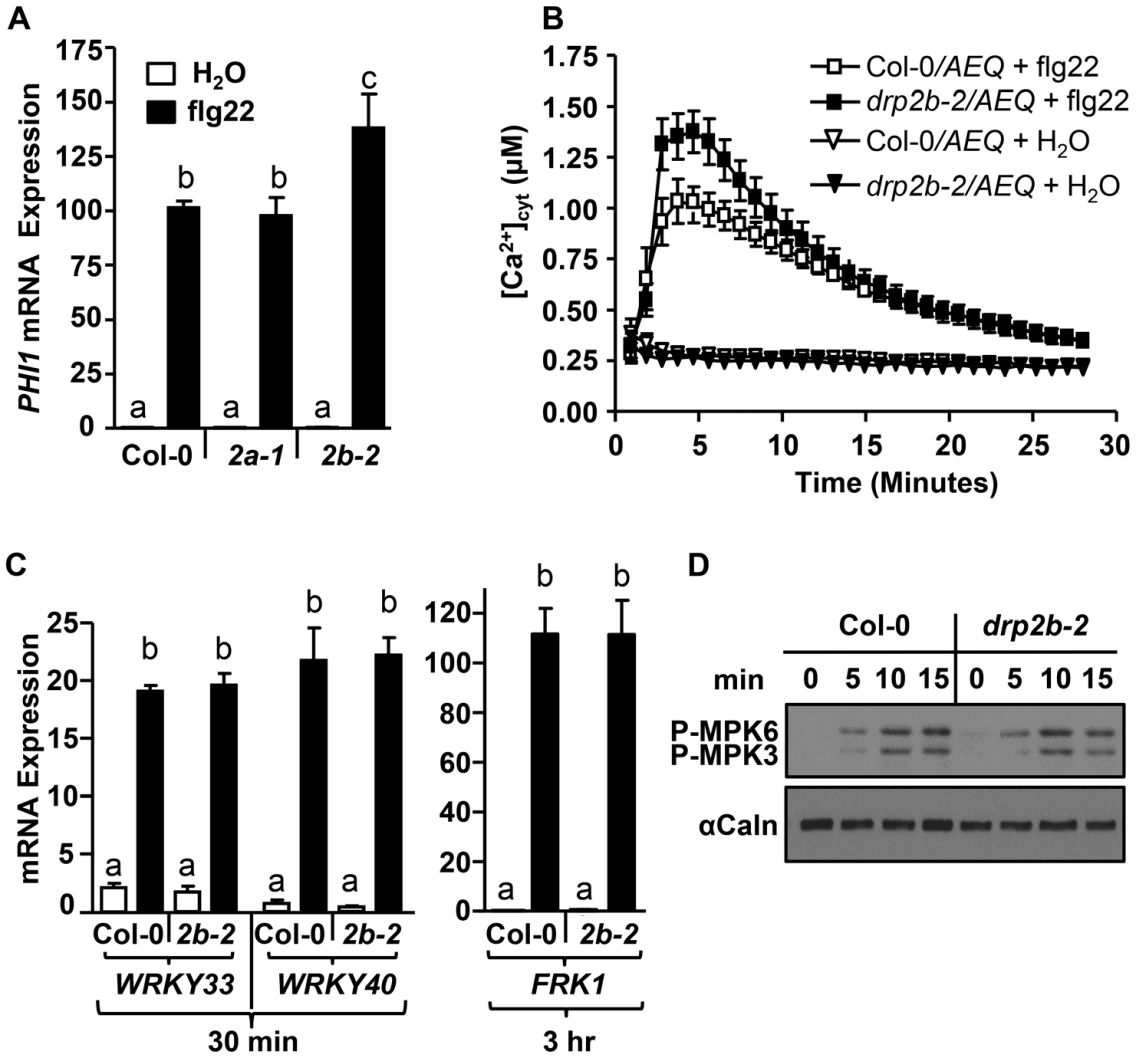
*DRP2B* is a negative regulator of flg22-induced Ca^2+^-dependent responses but has no apparent role in MAPK-dependent responses. (A) Using qRT-PCR, *PHI1* mRNA levels were increased in *drp2b-2* (*2b-2*; P<0.05), but not in *drp2a-1* (*2a-1*; P>0.5), compared to wild-type Col-0 at 30 min post-elicitation with 1 µM flg22 (black bars). Water treatment (white bars) at 30 min are mock control (P>0.5). (n = 3/genotype and treatment). (B) After 0.1 µM flg22 elicitation, cytosolic Ca^2+^ levels were significantly elevated in *drp2b-2* expressing the Ca^2+^-reporter Aequorin (*drp2b-2/*AEQ; closed square) compared to Col-0 expressing Aequorin (Col-0/AEQ; open square). Mock (H_2_O)-treatment served as control (triangles). (n = 6/genotype and treatment). (C) Using qRT-PCR, mRNA levels of *WRKY33, WRKY40* and *FRK1* were not significantly different between *drp2b-2* (*2b-2*) and Col-0 at indicated times after elicitation with water (white bars) or 1 µM flg22 (black bars). P-values for *WRKY33, WRKY40*, *FRK1* mRNA levels between *drp2b-2* and Col-0 were all P>0.5. (n = 3/genotype and treatment). (D) No apparent difference in flg22-induced phosphorylation of MPK3 and MPK6 was observed between Col-0 and *drp2b-2* (*2b-2*) after elicitation over 15 minutes (min) following elicitation with 0.1 µM flg22. Immunoblot analysis was done on total protein extracts probed with an antibody for phosphorylated MAPKs (P-MPK3 and P-MPK6). αCalnexin (αCaln) served as loading control. Experiments in (A,C and D) utilized 5–6 week old leave tissue, and experiments in (B) used cotyledons from 8-day old seedlings. All experiments were repeated at least 3 times with similar results. Values are mean ± SE. Statistical analysis was done as in Fig. 1.

To test whether *drp2b-2* displays other flg22-signaling defects, we measured flg22-induced changes in mRNA expression of *PHI1*, a Ca^2+^-dependent marker gene that is up-regulated in response to flg22 [19]. As determined by qRT-PCR (Fig. 2A), *PHI1* transcript accumulation was significantly increased in *drp2b-2* compared to Col-0 and *drp2a-1* at 30 minutes post-elicitation with flg22 (Fig. 2A). No statistically significant differences were detected between flg22-treated *drp2a-1* and Col-0 or between mock (H_2_0)-treated tissues (Fig. 2A). Because *drp2b-2* but not *drp2a-1* single mutant plants showed heightened flg22-induced ROS production and *PHI1* mRNA levels, we focused the remainder of this study on further characterizing the role of DRP2B in innate immune responses utilizing the *drp2b-2* mutant.

One of the earliest signaling events occurring within 1–2 minutes after PAMP perception is a rapid increase in [Ca^2+^]_cyt_ which in turn contributes to PAMP-induced ROS production and *PHI1* mRNA levels [19], [57]. To examine whether *DRP2B* also functioned in flg22-induced changes of [Ca^2+^]_cyt_, *drp2b-2* was crossed with a Col-0 plant line expressing the cytosolic calcium reporter aequorin (AEQ), a well-established photoreporter protein to assess changes in cellular calcium levels [41], [57]. When elicited with flg22, 8-day as well as five-to six-week old homozygous *drp2b-2/AEQ* plants displayed a highly significant increase in [Ca^2+^]_cyt_ compared to Col-0/*AEQ* (Fig. 2B and S2A Fig., respectively). Specificity of this response was demonstrated by the lack of increased [Ca^2+^]_cyt_ in mock (H_2_O)-treated samples (Fig. 2B). Pretreatment with LaCl_3_, a Ca^2+^ -channel blocker [57], [58], abolished flg22-induced [Ca^2+^]_cyt_ in both *drp2b-2/AEQ* and Col-0/*AEQ* to statistically similar levels (S2B Fig.). Similarly, no difference in flg22-induced ROS production was detected between *drp2b-2* mutant and wild-type plants after LaCl_3_ pretreatment (S2C Fig.). These LaCl_3_ results indicate that in *drp2b*, the flg22-elicited increase in [Ca^2+^]_cyt_ and ROS production was dependent on the activity of Ca^2+^-channel(s).

To further investigate the transcriptional reprogramming that occurs downstream of DRP2B, we measured the flg22-induced changes in mRNA expression of *PER62*, *PER4*, and *NHL10*, which are marker genes reported to be synergistically affected by CDPKs and MAPKs [19], as well as the MAPK-dependent marker genes *WRKY33*, *WRKY40* or *FRK1* [19], [59]. As determined by qRT-PCR comparing *drp2b-2* to Col-0, no significant differences were observed for transcript accumulation for any of these genes before or after flg22-treatment (S3 Fig., *PER62, PER4*, *NHL10*; Fig. 2C, *WRKY33*, *WRKY40, FRK1*). Furthermore, we probed total protein samples with an antibody that detects phosphorylated MAPKs [38] to examine activation of the MAPK-pathway in response to flg22. No statistically significant differences in flg22-induced phosphorylation of MPK3 and MPK6 were evident in *drp2b-2* compared to Col-0 between 0 to 45 min (Fig. 2D and S4A Fig. for 0 to 15 min; S4B and S4C Figs. for 0 to 45 min).

Taken together, our results reveal that loss of *DRP2B* differentially affected distinct branches of the flg22-signaling network. Specifically, we observed that *DRP2B* functioned as a negative regulator of early Ca^2+^-dependent responses while having no apparent role in MAPK-dependent signaling.

### Elevated flg22-induced ROS production in *drp2b* mutants is Wortmannin- but not Tyrphostin A23-sensitive

**Figure 3:**
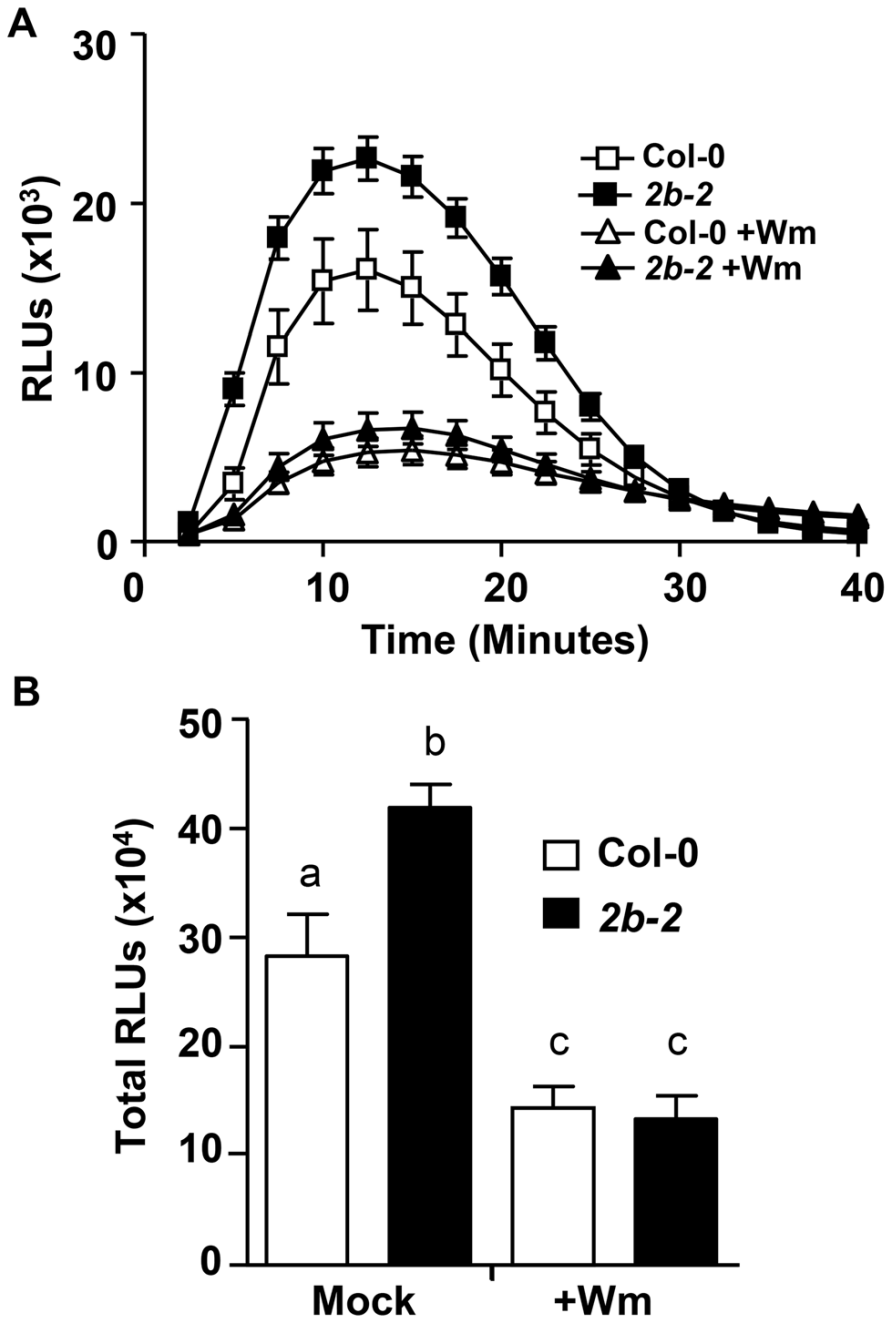
For *drp2b*, increased flg22-induced ROS production is sensitive to Wortmannin. (A) After a one-hour pretreatment in the absence (square) or presence (triangle) of 30 µM Wortmannin (Wm), ROS production was measured over 40 minutes in *drp2b-2* (*2b-2*, closed symbols) and Col-0 (open symbols) in response to 1 µM flg22 (n = 24/treatment and genotype). (B) Total ROS production based on time-course experiments shown in (A) indicates that in *drp2b-2* (*2b-2*, black bars), the increase in flg22-induced ROS production is Wm-sensitive (compare *2b-2*+Wm vs Col-0+Wm, P>0.5). Experiments were done using five-to-six week old leaf tissue and repeated at least three independent times with similar results. Values are mean ± SE. Statistical analysis was done as in Fig. 1. Relative Light Units, RLU.

Recently, we have shown that flg22-elicited ROS production but not MAPK phosphorylation is sensitive to chemical interference with the vesicular trafficking inhibitor Wortmannin (Wm) [13]. Wm is a well-established phosphatidylinositol (PI)-3- and PI-4-kinase inhibitor [13], which interferes with the maturation of late endosomes and multivesicular bodies [60], [61]. In plant cells, Wm also inhibits the formation of endocytic vesicles at the PM [62], thereby impeding ligand-induced endocytosis and degradation of FLS2 [9], [12], [13]. Given that both loss of *DRP2B* (Figs. 1 and 2) and Wm-treatment [13] affect flg22-elicited ROS production but not MAPK phosphorylation, we examined whether the increase in flg22-induced ROS production in *drp2b-2* was Wm-sensitive. Prior to flg22-elicitation, leaf tissue was pre-incubated for one hour with 30 µM Wm, a concentration previously shown to interfere with flg22-induced endocytic degradation of FLS2 [9], [13] and ROS production [13]. In agreement with our previous study [13], pretreating Col-0 leaf discs with Wm resulted in a significant decrease in flg22-induced ROS production compared to mock-treated wild-type tissue (Fig. 3A-B; compare mock and +Wm for Col-0). For *drp2b-2*, the inhibitor pretreatment also significantly decreased flg22-elicited ROS production (Fig. 3A-B; compare mock and +Wm for *drp2b-2*), and importantly, we consistently observed no statistically significant difference in flg22-elicited ROS levels between *drp2b-2* and Col-0 lines following Wm treatment (Fig. 3A-B; compare +Wm for *drp2b-2* and Col-0).

Next, we tested whether in *drp2b-2*, the increase in flg22-induced ROS is sensitive to chemical interference with Tyrphostin A23 (TyrA23). TyrA23 was initially identified as a tyrosine kinase inhibitor [63]; however, it also functions as a vesicular trafficking inhibitor by interfering with the internalization of endocytic vesicles from the PM [63]–[68]. We have recently shown that similar to Wm, pretreatment with TyrA23 results in reduced flg22-elicited ROS production in wild-type plants [13]; but importantly, reduced ROS levels can be attributed to its function as a Tyr kinase inhibitor rather than its role as a vesicular trafficking inhibitor [13]. When pretreating leaf tissue with 100 µM TyrA23, flg22-induced ROS production was significantly decreased in both *drp2b-2* and Col-0 leaf tissue (S5A and S5B Figs.; compare –T23 vs +T23 for either Col-0 or *drp2b-2;* P<0.0001) indicating that this inhibitor was functional. However, when comparing ROS levels between mutant and wild-type in the presence of TyrA23, we consistently observed that flg22-induced ROS production was still significantly increased in *drp2b-2* compared to Col-0 (S5A and S5B Figs.; compare +T23 between *drp2b-2* and Col-0; P≤0.025). Taking these chemical interference results together, DRP2B's role as a negative regulator in flg22-induced ROS production appears to be part of a signaling pathway that is Wm- but not fully TyrA23-sensitive.

### Loss of *DRP2B* differentially affects late flg22-responses in a non-canonical manner

**Figure 4:**
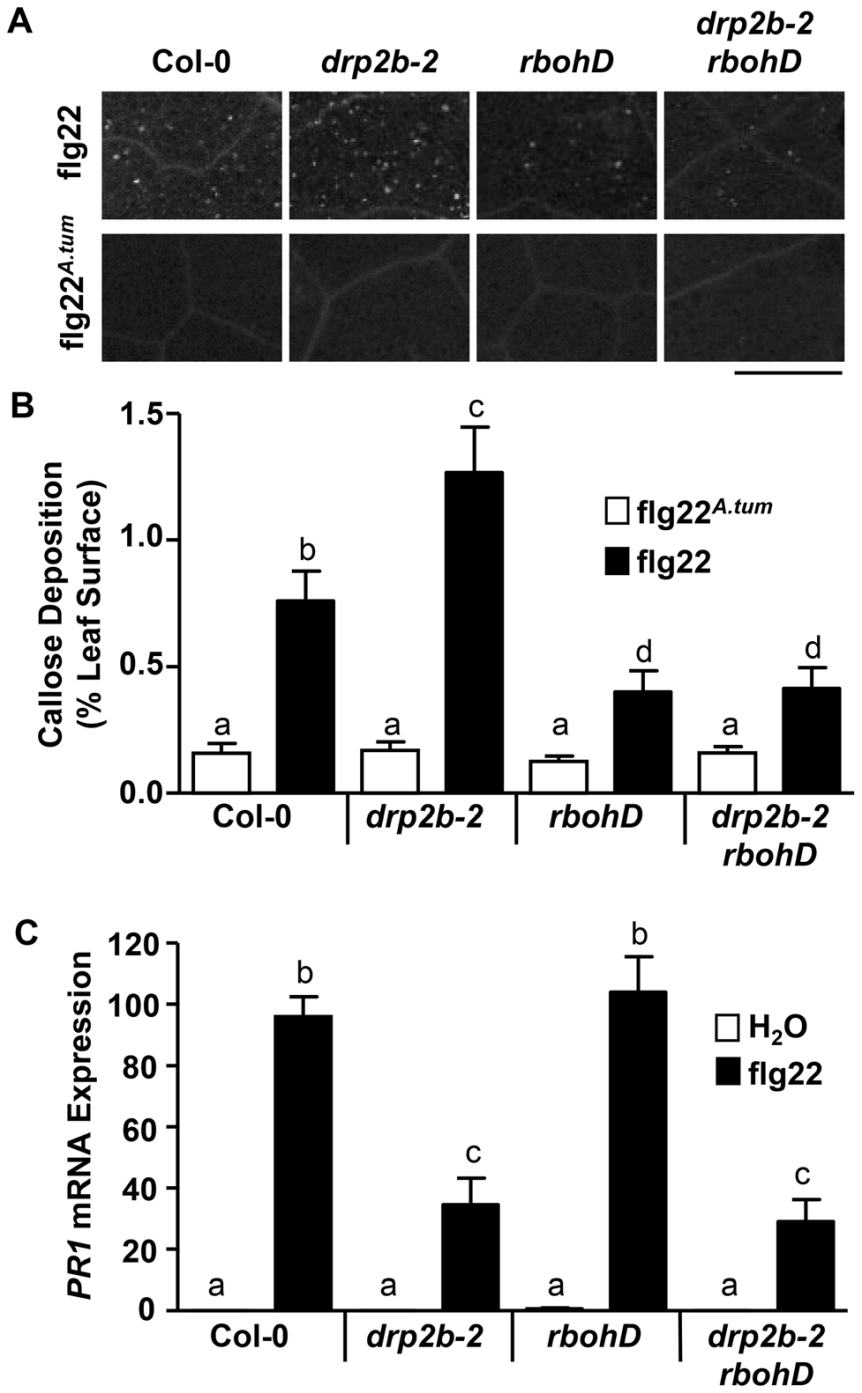
Loss of *DRP2B* affects late flg22-responses in a non-canonical manner. (A) Callose deposition in five-to-six week old leaf tissue of Col-0, *drp2b-2*, *rbohD*, and *drp2b-2 rbohD* at 24 hr after infiltration of 10 nM flg22 or 10 nM flg22*^A.tum^*. A representative image is shown depicting differences in callose depositions between genotypes and treatments. Scale bar = 0.5 mm. (B) Percentage of total leaf surface area covered by aniline blue-stained fluorescent callose at 24 hr after infiltration of 10 nM flg22 (black bars) or 10 nM flg22*^A.tum^* (white bars). (n>20/genotype and treatment). (C) Using qRT-PCR, *PR1* mRNA levels were significantly reduced in *drp2b-2* and *drp2b-2 rbohD* compared to Col-0 (P<0.0001) or *rbohD* (P<0.0001) at 24 hrs after infiltration with 1 µM flg22 (black bars). Treatment with water (white bars) served as mock control (P>0.5). (n = 4/genotype and treatment). All experiments were done using five-to-six week old leaf tissue and repeated at least three independent times with similar results. Values are mean ± SE. Statistical analysis was done as in Fig. 1.

To investigate whether loss of *DRP2B* affects late flg22-responses, callose, a β-1,3-glucan polymer deposited at the cell wall in response to flg22, was visualized by aniline blue staining. When leaves of mature plants were infiltrated with active flg22, *drp2b-2* showed a significant increase in flg22-induced callose deposits compared to Col-0 at 24 hours post-infiltration (Figs. 4A-B). In control experiments, no difference was detected in response to inactive flg22*^A^* ^.*tum*^ (Fig. 4A-B) indicating that in *drp2b-2*, the increased number of callose deposits was not due to a wound-effect or pre-existing callose deposits. Because flg22-induced callose deposition requires functional *RbohD* [48], [56], we utilized the *drp2b-2 rbohD* double mutant to examine whether in *drp2b-2*, the increase in flg22-elicited callose deposition was *RbohD*-dependent. A significant decrease in flg22-induced callose deposition was observed in both *rbohD* single and *drp2b-2 rbohD* double mutants compared to Col-0 and *drp2b-2* (Figs. 4A-B). Importantly, no statistical difference was observed between *rbohD* and *drp2b-2 rbohD* (Fig. 4B) indicating that *DRP2B* functions as a negative regulator of flg22-induced callose deposition in a *RbohD*-dependent manner, and that in *drp2b-2*, increased callose deposition may be a downstream result of enhanced *RbohD-*dependent ROS production.

Expression of *Pathogen-Related 1* (*PR1*), a commonly used late marker gene typically associated with SA-regulated signaling responses, is up-regulated after flg22-treatment or pathogen infection [45]. After twenty-four hours elicitation with flg22, but not with mock (H_2_O) treatment, an up-regulation of *PR1* mRNA levels was observed for both *drp2b-2* and Col-0. However, the increase in *PR1* mRNA expression level was significantly reduced in *drp2b-2* compared to Col-0 as determined by qRT-PCR (Fig. 4C). Because *RbohD* has been proposed to function as a negative regulator of SA-responses [21], [69], we examined whether the decreased flg22-induced *PR1* expression in the *drp2b-2* mutant was due to an increase in *RbohD*-dependent responses. As shown in Fig. 4C, flg22-elicited *PR1* mRNA expression levels in *rbohD* were similar to Col-0. *PR1* mRNA levels in the *drp2b-2 rbohD* double mutant, however, were not restored to those measured in *rbohD* but were similar to those in *drp2b* (Fig. 4C). Thus, we conclude that in *drp2b-2*, the increased *RbohD*-dependent responses are unlikely to be responsible for diminished levels of flg22-induced *PR1* mRNA expression.

To test whether decreased *PR1* mRNA levels in *drp2b* were due to altered Ca^2+^-channel activity, we co-treated plants with 1 µM flg22 and 10 mM LaCl_3_ for 24 hours prior to qRT-PCR analysis. Compared to flg22 elicitation alone, LaCl_3_ co-treatment resulted in a significant decrease in flg22-induced *PR1* mRNA levels in both Col-0 and *drp2b-2* (S6 Fig.) indicating that robust transcriptional activation of *PR1* required activity of Ca^2+^ channel(s). But importantly, *PR1* mRNA levels were still significantly decreased in *drp2b-2* compared to Col-0 under these co-treatment conditions (S6 Fig.). Based on these results, we conclude that *DRP2B* functions as a positive regulator of flg22-induced *PR1* mRNA expression that was partially independent of Ca^2+^-channel activity. Thus, DRP2B's role in modulating *PR1* mRNA levels appears to be different from its role as a negative regulator in ROS production and callose deposition, which were dependent on both *RbohD* and Ca^2+^-channel activities.

### *drp2b* shows increased ROS production and decreased *PR1* mRNA in response to living *Pto* strains in a Type 3 Secretion System-independent manner

**Figure 5:**
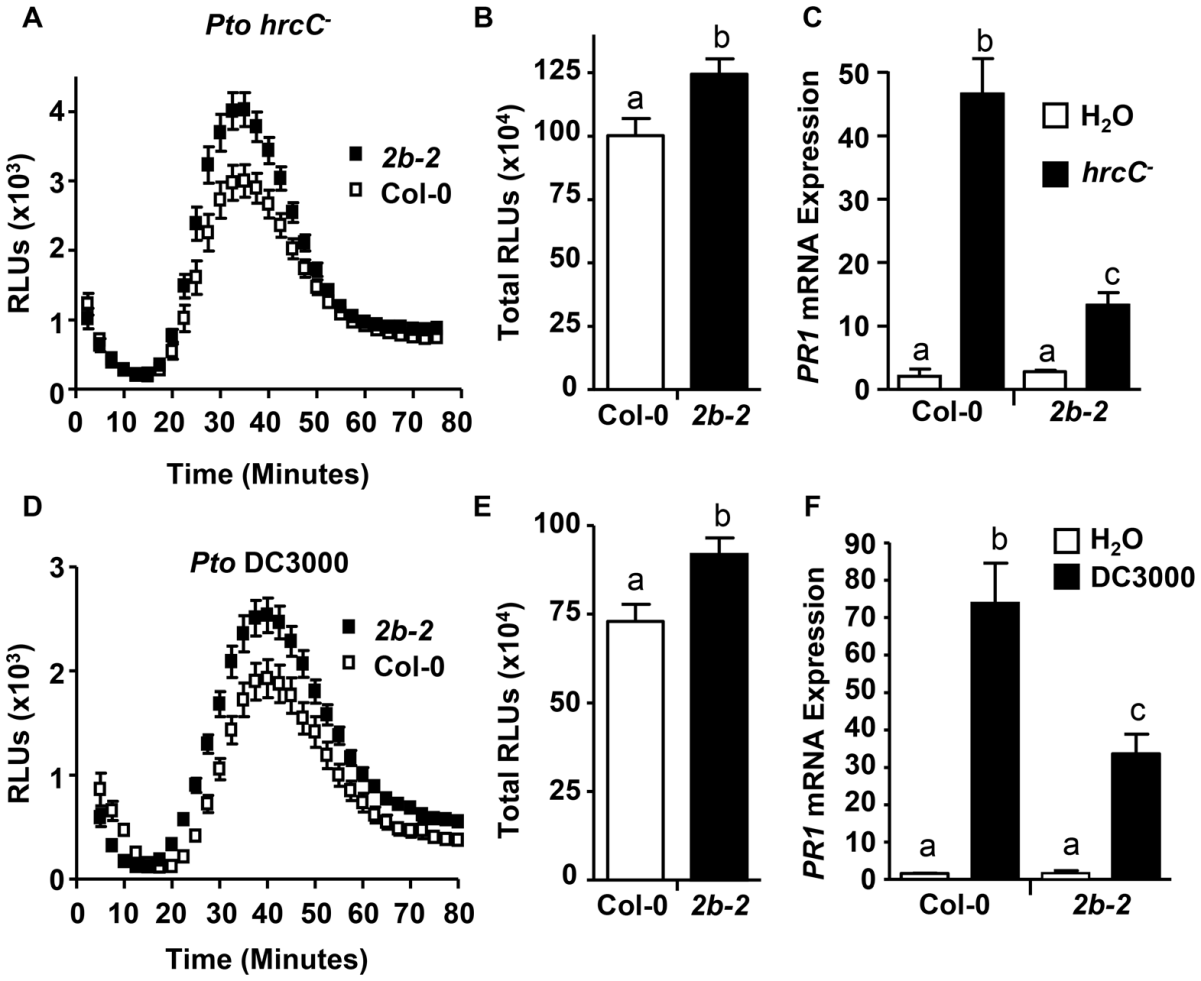
*drp2b* shows increased ROS and decreased *PR1* mRNA to living *Pto* in a T3SS-independent manner. (A) and (D) Time-course of ROS production in Col-0 (open symbol) and *drp2b-2* (*2b-2*; filled symbol) in response to living (A) *Pto hrcC^-^* or (D) *Pto* DC3000 (OD_600_ = 0.1). (n = 32/genotype). (B) and (E) Compared to Col-0, total ROS production was significantly increased in *drp2b-2* (*2b-2*) after elicitation with (B) *Pto hrcC^−^* (P<0.01) or (E) *Pto* DC3000 (P<0.007) based on values shown in (A) or (D), respectively. Relative Light Units, RLU. (C) and (F) Using qRT-PCR, *PR1* mRNA levels were significantly reduced in *drp2b-2* (black bars) compared to Col-0 (white bars) at 24 hr after infiltration with (C) *Pto hrcC^−^* (OD_600_ = 0.02) (P<0.045) or (F) *Pto* DC3000 (DC; OD_600_ = 0.02) (P<0.005). Infiltration with water (white bars) served as mock control. (n = 6/genotype and treatment). All experiments were done using five-to-six week old leaf tissue and repeated at least three independent times with similar results. Values are mean ± SE. Statistical analysis was done as in Fig. 1.

Next, we investigated whether DRP2B's role as a negative regulator for ROS production but as positive a regulator for *PR1* mRNA levels was specific to flg22 or whether its regulatory roles may be also observed in response to flagellated bacterial *Pto* strains. Live *Pto* cells elicit an early and transient ROS production that is independent of the Type 3 Secretion System (T3SS) and is fully dependent on *RbohD* and *FLS2* [42]. Utilizing this ROS bioassay, ROS production was measured in response to the live *Pto* DC3000 *hrcC^−^* (*Pto hrcC^−^*) that elicits PAMP-dependent responses; but *Pto hrcC^−^* lacks a functional T3SS, and thus in contrast to the virulent bacterial strain *Pto* DC3000, *Pto hrcC^−^* is defective in effector delivery and cannot suppress Pattern-Triggered Immunity (PTI) [70]. For *drp2b-2*, treatment with *Pto hrcC^−^* resulted in increased ROS production including increased peak ROS (Fig. 5A; around 40 minutes post-treatment) as well as total ROS production (Fig. 5B) compared to Col-0. Similar results were obtained when treating *drp2b-2* leaf discs with *Pto* DC3000 (Fig. 5D-E).

For measuring *PR1* mRNA levels, *drp2b-2* mutant and wildtype leaves were syringe-infiltrated with *Pto hrcC^−^* or *Pto* DC3000. As determined by qRT-PCR, *PR1* mRNA levels were significantly induced by either bacterial strain for both *drp2b-2* and Col-0 compared to mock-treated tissue at 24 hours after infiltration; but the *Pto*-induced expression of *PR1* was significantly lower in *drp2b-2* than in Col-0 in response to *Pto hrcC^−^* or *Pto* DC3000 (Fig. 5C or 5F, respectively). Thus consistent with the flg22 results, *DRP2B* is a negative regulator of ROS production but a positive regulator of *PR1* mRNA levels in response to live bacterial *Pto* strains in a T3SS-independent manner.

### Consistent with decreased *PR1* mRNA levels, *drp2b* plants are more susceptible to *Pto* DC3000 infection

**Figure 6:**
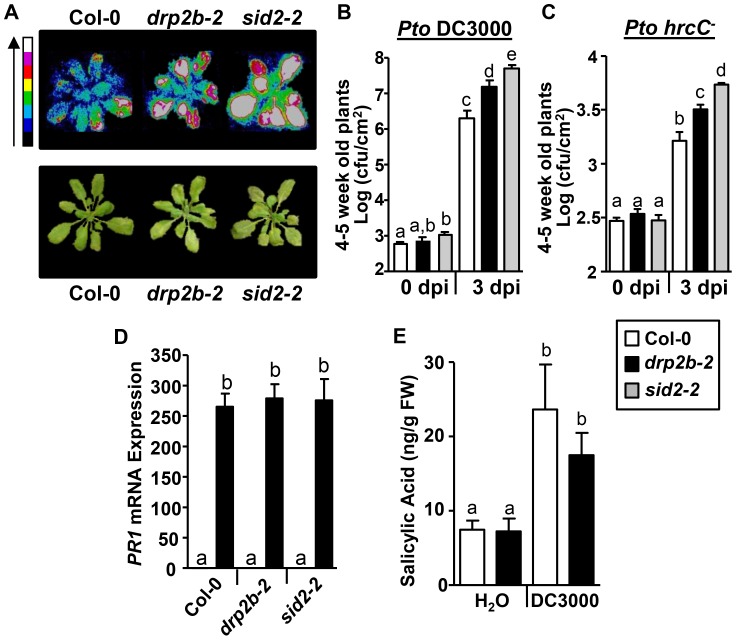
*drp2b* single mutants are more susceptible to *Pseudomonas syringae* pv. *tomato* (*Pto*) DC3000 and *Pto hrcC^−^* infection. (A) *In planta* growth of *Pto* DC3000 *LuxCDABE* (DC3000lux) in 4–5 week old Col-0, *drp2b-2* (*2b-2*), and *sid2-2* plants. Plants were imaged at 3 days post infiltration (dpi) after syringe-infiltration with DC3000lux (OD_600_ = 0.0005) by bright field or with a Photek camera. A representative plant is shown for each genotype and treatment. Color scale bar indicates increasing photon intensity. (n = 4 plants/genotype and treatment). (B) Compared to Col-0 (white bars), bacterial growth was significantly increased in *drp2b-2* plants (black bar) (P<0.0065) at 3 dpi with DC3000lux (OD_600_ = 0.0005) as measured by serial dilution plating. *sid2-2* served as control (gray bars; *sid2-2* to Col-0: P<0.0001; to *drp2b-2*: P<0.02). (n = 7-12/genotype and treatment). (C) Compared to Col-0 (white bars), bacterial growth was significantly increased in *drp2b-2* plants (black bar) (P<0.01) at 3 dpi with *Pto hrcC^-^* (OD_600_ = 0.02) as measured by serial dilution planting. *sid2-2* served as control (gray bars; *sid2-2* to Col-0: P<0.0001; to *drp2b-2*: P = 0.0005). (n = 6/genotype and treatment). (D) Using qRT-PCR, no difference in *PR1 mRNA* levels was observed between *drp2b-2, sid2-2* and Col-0 24 hrs after treatment with 50 µM exogenous SA (black bars). Treatment with water (H_2_O; white bars) served as mock control. (n = 4/genotype an treatment). (E) Levels of SA in *drp2b-2* (black bars) compared to Col-0 (white bars) at 24 hr post infection (hpi) with *Pto* DC3000lux (OD_600_ = 0.02) (P = 0.375). Treatment with water (H_2_O; white bars) served as mock control (P = 0.91). (n = 4/genotype and treatment; with each n containing 6 seedlings). Experiments in (A-C) and in (D-E) utilized 4–5 week or 2 week old plants, respectively. All experiments were repeated three times with similar results. Values are mean ± SE. Statistical analysis was done as in Fig. 1.

To examine whether the observed defects in flg22 and *Pto*-induced signaling responses in *drp2b-2* correlated with changes in resistance to these bacterial pathogens, we monitored the growth of *Pto* DC3000 strains after infiltration into mature five-to-six week old leaves. First, we utilized *Pto* DC3000 that stably expresses the *luxCDABE* operon (*Pto* DC3000*lux*) [44] allowing for the visualization of growth of this bioluminescent bacterial strain *in planta* using a Photek luminescent camera [20], [46]. Three days post-infection (3 dpi) with syringe-infiltrated *Pto* DC3000*lux, drp2b-2* exhibited increased bacterial growth compared to Col-0 (Fig. 6A); but *drp2b-2* appeared consistently less susceptible than *sid2-2* plants known to be highly susceptible to *Pto* DC3000*lux* infection because they do not express functional SID2 (SALICYLIC ACID INDUCTION-DEFICIENT 2), a key enzyme in defense-related SA biosynthesis [40]. These results were confirmed by bacterial serial dilution plating assays in that bacterial growth was significantly higher in *drp2b-2* than in Col-0 but lower than in *sid2-2* at 3 dpi (Fig. 6B). No statistical difference in bacterial growth was observed between *drp2b-2* and Col-0 or *sid2-2* leaves at 0 dpi (Fig. 6B). Similar results were obtained when *drp2b-2* leaves were infiltrated with *Pto hrcC^−^* (Fig. 6C), and this increased susceptibility of *drp2b-2* to *Pto hrcC^−^* was consistent with a function of *DRP2B* in PTI.

In complementary approaches, plants were grown for two weeks on MS plates and then were fully immersed in a solution of *Pto* DC3000*lux* [46] for pathogen infection assays. At this developmental stage, no significant difference in weight was observed between the *drp2b-2, Col-0* and *sid2-2* plant lines (S7A Fig.). Similar to phenotypic defects observed in five-to-six week old mature *drp2b-2* plants (see Figs. 1C-F and 4C), two week old *drp2b-2* plants exhibited increased ROS production and reduced *PR1* transcript accumulation in response to flg22 (S7B-D Fig.) as well as increased Pto DC3000*lux* susceptibility and decreased *PR1* transcript accumulation in response to *Pto* DC3000*lux* compared to Col-0 (S7E-F Fig.).

To gain insight into whether decreased *PR1* levels and increased susceptibility in *drp2b-2* may be due to defects in SA perception or accumulation, we first measured *PR1* mRNA levels after exogenous application of 50 µM SA to 2-week-old plants. As determined by qRT-PCR, *drp2b-2* accumulated similar levels of *PR1* mRNA levels compared to Col-0 and *sid2-2* (Fig. 6D) indicating that SA perception remained intact in *drp2b-2* plants. Next, we measured SA levels (ng/fresh weight) in 2-week-old *drp2b-2* and Col-0 plants 24 hpi with *Pto* DC3000*lux*. No significant difference in SA levels was observed between *drp2b-2* and Col-0 after treatment with *Pto* DC3000*lux* (Fig. 6E). Likewise, no significant difference in SA levels was observed between *drp2b-2* and Col-0 after treatment with flg22 (S7G Fig.). Because SA accumulation was measured under the same experimental conditions as *PR1* gene induction and resistance to *Pto* (S7D-F Fig.), we conclude that the increased susceptibility to *Pto* DC3000 in *drp2b-*2 appears to occur independently of SA accumulation.

### Robust ligand-induced endocytosis of FLS2 requires *DRP2B* but not *DRP2A*

Based on the subcellular localization of DRP2B to sites of CME from the PM and its role in constitutive endocytosis of yet unknown cargo [27], [33], DRP2B may modulate the trafficking of PM-resident proteins involved in flg22-signaling. FLS2 is a likely candidate for DRP2B-dependent trafficking because FLS2 undergoes ligand-induced endocytosis and subsequent degradation at 60 minutes post-elicitation [9], [12], [13].

**Figure 7:**
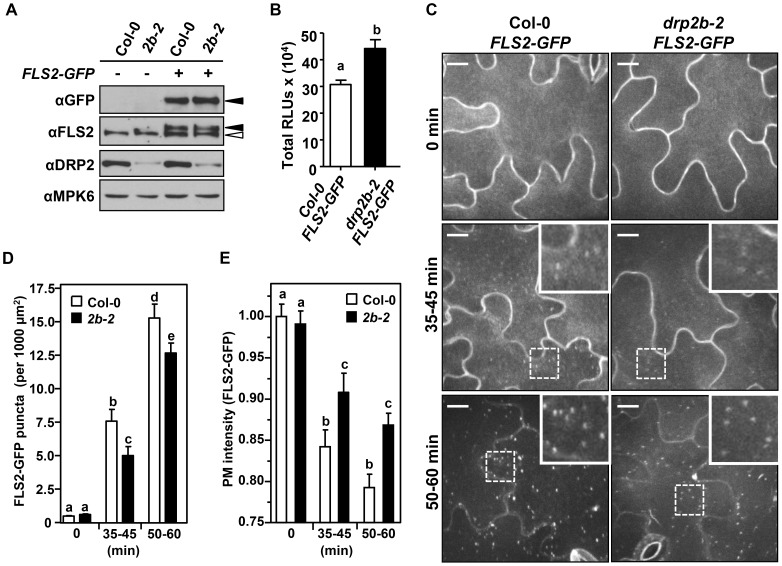
DRP2B is partially required for flg22-induced endocytosis of FLS2. (A) Col-0 *FLS2-GFP* and *drp2b-2 FLS2-GFP* (*2b-2*) homozygous F4 seedlings expressed similar levels of both endogenous FLS2 and FLS2-GFP as shown by immunoblot analyses of total protein extracts. αFLS2 detected both native FLS2 (open arrow) and FLS2-GFP (closed arrow) while αGFP detected FLS2-GFP only (closed arrow). αDRP2 was used to confirm *drp2b-2* mutants, and αMPK6 was used as a loading control. (B) In response to 1 µM flg22, total ROS production was elevated in *drp2b-2 FLS2-GFP* (black bar) compared to Col-0 *FLS2-GFP* (white bars) cotyledons (P<0.001). (n = 30 cotyledons/genotype). Relative Light Units, RLU. (C) Flg22-induced endocytosis of FLS2-GFP was not blocked in *drp2b-2* cotyledons. Col-0 *FLS2-GFP* (Col-0) and *drp2b-2 FLS2-GFP* whole seedlings were treated with 1 µM flg22 to observe un-elicited (constitutive; 0 min) and ligand-induced (35–45, 50–60 min) endocytosis of FLS2-GFP by spinning disc confocal microscopy. Representative maximum-intensity projection images and zoomed insets of FLS2-GFP fluorescence are shown, with bright pixels corresponding to increased abundance of FLS2-GFP at a given location. Scale bars = 10 µm. (D) Quantification of FLS2-GFP in puncta at 0, 35–45 or 50–60 min after elicitation with 1 µM flg22 indicates that loss of *DRP2B* resulted in ∼20% decrease in flg22-stimulated endocytosis of FLS2-GFP (35–45 min, P = 0.0204; 50–60 min, P = 0.0396). No change in unstimulated accumulation of FLS2-GFP in puncta (P>0.05, 0 min) was observed. Images from two independent experiments were included in the analysis. (n = 44 to 67 images analyzed per genotype/treatment). (E) In *drp2b-2* cotyledons, decreased accumulation of FLS2-GFP in puncta correlates with increased PM intensity of FLS2-GFP after elicitation with 1 µM flg22 relative to Col-0 (35–45 min, P = 0.0302; 50–60 min, P = 0.0001). No significant differences are observed in the PM intensity of FLS2-GFP in unstimulated Col-0 and *drp2b-2* cotyledons (P>0.05, 0 min). Images from 2–3 independent experiments were included in the analysis. (n = 76–151 images analyzed per genotype/treatment). All experiments were repeated at least three independent times with similar results. Values are mean ± SE. Statistical analysis was done as in Fig. 1.

To detect potential spatial and temporal defects in FLS2 localization, we crossed Col-0 plants expressing *FLS2pro:FLS2-3xMyc-EGFP* [9] with *drp2b-2* plants to generate homozygous Col-0 *FLS2-GFP* and *drp2b-2 FLS2-GFP* sibling plants for live cell imaging studies. To ensure equal expression of FLS2-GFP in both Col-0 and *drp2b-2*, we performed immunoblot analyses of un-elicited seedlings under conditions grown and prepared for microscopy. Similar to *drp2b-2* mutants, *drp2b-2* FLS2-GFP seedlings displayed reduced levels of DRP2 protein (Fig. 7A, αDRP2). Consistent with Fig. 1G, similar levels of endogenous FLS2 were detected between Col-0 *FLS2-GFP* and *drp2b-2 FLS2-GFP* plants (Fig. 7A; αFLS2). Importantly, equivalent expression was also observed for GFP-tagged FLS2 in Col-0 and *drp2b* seedlings (Fig. 7A; αGFP). As observed in *drp2b-2* which expressed endogenous FLS2 but not FLS2-GFP (Fig. 1D), *drp2b-2* FLS2-GFP cotyledons displayed increased flg22-induced ROS production compared to Col-0 FLS2-GFP (Fig. 7B). This result indicates that ectopic FLS2-GFP expression did not abrogate the difference in flg22-induced ROS production between Col-0 and *drp2b-2*. Therefore, FLS2-GFP cotyledons can be used as a biologically relevant tissue for microscopic analysis of the role of DRP2B in FLS2 trafficking.

Next, Col-0 FLS2-GFP and *drp2b-2* FLS2-GFP seedlings were used for quantitative live cell imaging using Spinning Disc Confocal Microscopy (SCDM), a technique previously utilized to quantify ligand-induced endocytosis of FLS2-GFP in *A. thaliana* [9], [14]. First, we confirmed that under un-elicited conditions, FLS2-GFP resided primarily at the PM of pavement cells on the adaxial surface of both Col-0 and *drp2b-2* cotyledons (Fig. 7C, 0 min) with detection of some FLS2-GFP in intracellular compartments (Fig. 7D, 0 min) as determined by quantification of the number of FLS2-GFP-positive puncta (per 1000 µm^2^ area) within cells. In addition, we detected equivalent levels of FLS2-GFP at the PM in un-elicited tissues based on PM intensity measurements (Fig. 7E, 0 min), indicating that altered PM abundance of FLS2 was not a likely cause of early flg22-reponse defects in *drp2b*. Consistent with previous reports showing ligand-induced internalization of FLS2-GFP and subsequent movement through early and late endosomal compartments that appear as FLS2-GFP-containing puncta [9], [10], [12], [14], elicitation of Col-0 with 1 µM flg22 led to significant internalization of FLS2-GFP into endosomal compartments at 35–45 min and 50–60 min (Fig. 7C-D; Col-0). Endosomal accumulation of FLS2-GFP coincided with a significant decrease in FLS2-GFP detected at the PM (Fig. 7E; Col-0). Similar timing of FLS2-GFP accumulation in endosomes was observed for *drp2b-2* (Fig. 7C-D; 35–45 min and 50–60 min, *drp2b-2*); but importantly, quantification of the number of FLS2-GFP-positive endosomes (puncta per 1000 µm^2^ area) after flg22-elicitation showed a significant and consistent 20% decrease in *drp2b-2* compared to Col-0 (Fig. 7D). This reduction correlated with a significant decrease in removal of FLS2-GFP from the PM in *drp2b-2* as determined by PM intensity measurements (Fig. 7E; compare *drp2b-2* with Col-0 at 35–45 and 50–60 min). Combined, these results implicate DRP2B in ligand-induced endocytosis of FLS2.

To test for specificity in flg22-induced endocytosis of FLS2 within the DRP2 protein family, we generated *drp2a-3 FLS2-GFP* and sibling Col-0 *FLS2-GFP* plants for live-cell imaging that express equivalent levels of endogenous FLS2 and FLS2-GFP (S8A Fig.). First, we confirmed that as for *drp2a* mutant plants not expressing FLS2-GFP (Figs. 1D-E), *drp2a-3 FLS2-GFP* cotyledons did not show any significant difference in flg22-induced ROS production compared to Col-0 *FLS2-GFP* (S8B Fig.). Importantly, as determined by quantitative live cell imaging, no significant differences in the number of FLS2-GFP endosomes were observed in *drp2a-3* compared to Col-0 in un-elicted (S8C-D Fig., 0 min) or flg22-elicted cotyledons (S8C-D Fig., 50–60 min), indicating that *DRP2A* plays no apparent role in ligand-induced internalization of FLS2-GFP.

In conclusion, these live-cell imaging results (Fig. 7 and S8 Fig.) indicate that FLS2 undergoes flg22-induced endocytosis that is partially dependent upon DRP2B, but not the close homolog DRP2A.

## Discussion

**Figure 8:**
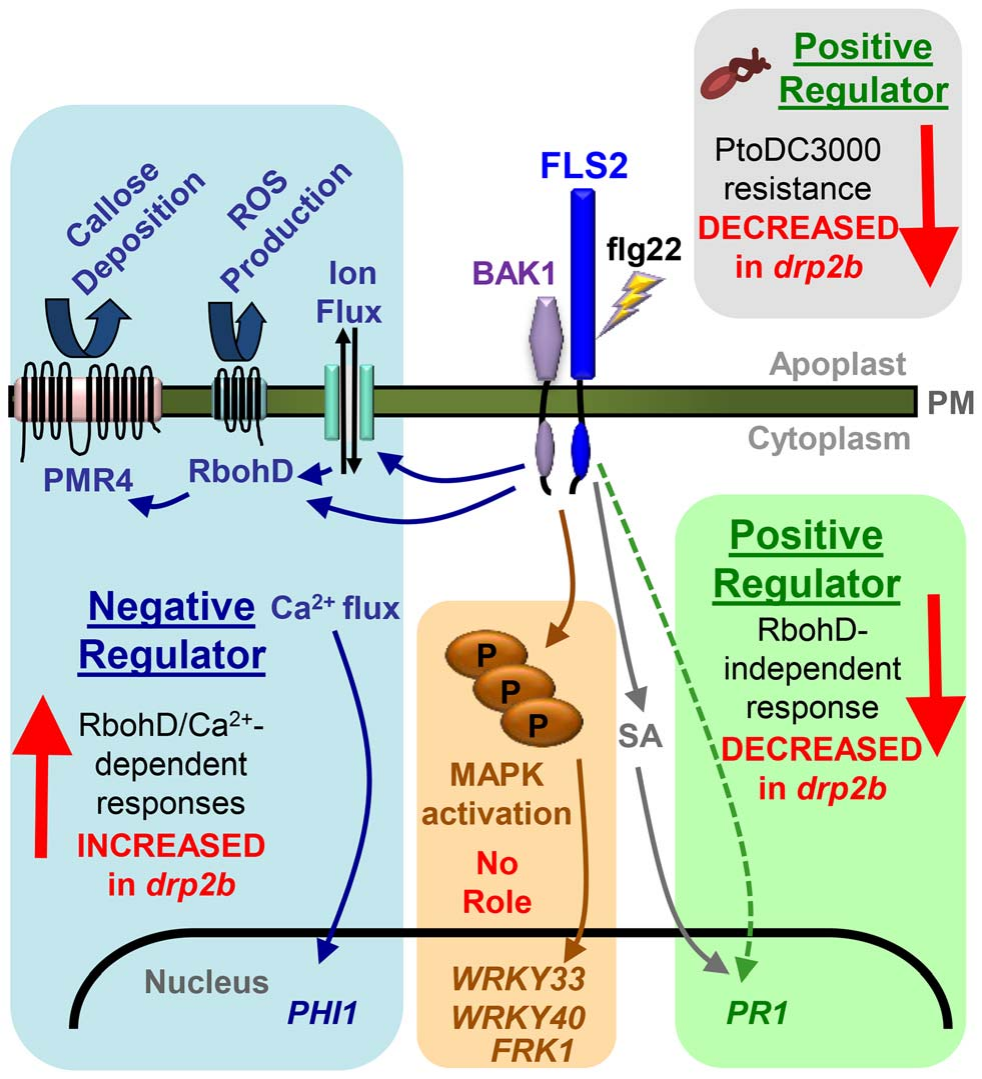
Summary of non-canonical response defects in *drp2b* within the different branches of the flg22-signaling network. Flg22-binding to FLS2 and its co-receptor BAK1 initiates at least three distinct branches of flg22-signaling networks that are differentially affected by loss of *DRP2B* compared to wildtype plants. All tested *RbohD*/Ca^2+^-dependent responses are increased in *drp2b* plants (blue box) consistent with DRP2's function as a negative regulator of these responses. Loss of *DRP2B* has no effect upon flg22-induced MAPK pathway activation (brown box) implying that DRP2B has no apparent role in the MAPK pathway. However, *drp2b* displays decreased *PR1* mRNA expression in response to flg22, which occurs independently of *RbohD* (green box) and may be at least in part, independent of SA. Correlating with decreased transcription of *PR1*, *drp2b* plants show decreased resistance to bacterial pathogen infection (*Pto* DC3000) (gray box) indicating that DRP2B is a positive regulator of resistance to this bacterial strain. The non-canonical combination of phenotypic defects observed in *drp2b* may be in part due to altered vesicular trafficking of FLS2 and potentially other yet unknown PM-resident cargo proteins. For simplicity, not all known components of the flg22-signaling network are included in this model.

Here, we identified DRP2B, previously implicated as a CCV component with roles in CME in plants [33], [34], as a novel factor functioning in flg22-signaling and innate immunity against *Pto* DC3000 and *Pto hrcC^-^*. We also provide evidence for a role of DRP2B in ligand-induced endocytosis of the plant flagellin receptor FLS2. As summarized in Fig. 8, loss of *DRP2B* revealed separation of innate immune signaling responses into at least three distinct branches of the flg22-signaling network that differ in their requirement for the NADPH oxidase RbohD.

### Role of DRP2B in distinct immune signaling branches

Our results strongly support the emerging conceptual change in the field of plant immunity that flg22-signaling events occur through a signaling network rather than a single linear pathway. More specifically, our results discovered that DRP2B has roles as a negative regulator of flg22-induced cytosolic Ca^2+^ levels, ROS production, *PHI1* mRNA levels and callose deposition (Figs. 1C-E, 2A-B and 4A-B). In light of our results and other studies [19], [48], [56], these signaling responses can be placed into the same branch of the flg22-signaling network, which we refer to as the *RbohD*/Ca^2+^-dependent branch (Fig. 8). Activation of RbohD has been reported to occur via Ca^2+^-dependent and independent mechanisms [19], [58], [71]–[73]; but our LaCl_3_ study indicate that for *drp2b*, the flg22-elicited increase in cytosolic Ca^2+^ was fully responsible for the increase in ROS production (S2 Fig.). The identification of a DRP2B peptide as an *in vitro* substrate for Ca^2+^-dependent protein kinases (CDPKs) provides further evidence of a link between DRP2B and the Ca^2+^-dependent response pathway(s) [74].

In contrast to its role as a negative regulator in the *RbohD*/Ca^2+^-signaling branch, DRP2B functions as a positive regulator of *PR1* mRNA levels in response to flg22 and to *Pto* in a T3SS-independent manner (Figs. 4C, 5C and 5F). Further support for the proposed separation of these signaling pathways into two distinct flg22-signaling branches (Fig. 8) was provided by assessing the genetic requirement of *RbohD* for specific flg22-responses. Increases in flg22-induced ROS production and callose deposition observed in *drp2b* were *RbohD*-dependent (Figs. 1F and 4B). In contrast, the decrease in flg22-induced *PR1* mRNA accumulation, a commonly used marker gene for SA responses, was *RbohD*-independent (Fig. 4C). If RbohD were to function as a negative regulator of SA-pathway responses within PAMP-triggered immunity as previously proposed [21], [69], then a rescue to wildtype *PR1* mRNA levels in *drp2b rbohD* plants would have been expected after flg22-treatment. Instead, both *drp2b* and *drp2b rbohD* plants showed a similarly reduced induction of *PR1*, indicating that component(s) other than RbohD may function as a negative regulator(s) of *PR1*/SA-responses. While the inability of *drp2b* plants to induce wildtype levels of *PR1* mRNA correlates well with its increased susceptibility to *Pto* DC3000 and *Pto hrcC^-^* (Figs. 5C, 5F and 6A-C), these phenotypic defects appear to be mostly independent or downstream of SA perception and accumulation (Fig. 6D-E). Consistent with the latter, a recent study dissecting signaling networks triggered by pathogenic *Pto* strains provides evidence for a separation of *PR1* induction and SA accumulation [75]. Furthermore, in response to a pathogenic *Pseudomonas* strain, the ARF-guanine nucleotide exchange factor (ARF-GEF) AtMIN7 has been recently reported to positively regulate *PR1* expression via a SA-independent pathway as a *atmin7* mutant displays reduced *PR1* expression without a significant reduction in SA accumulation [76]. However in response to flg22, loss of *AtMIN7* results in decreased callose deposition but has no effect on *PR1* levels [77], indicating that AtMIN7 and DRP2B likely have distinct roles in flg22-signaling.

DRP2B did not appear to play any significant role in modulating the MAPK pathway (MPK6 and MPK3 phosphorylation, upregulation of *WRKY33, WRKY40* and *FRK1* mRNA levels; Fig. 2C-D and S4 Fig.), which comprises the third branch of the flg22-signaling network in our model (Fig. 8). Thus, our findings provide independent support of recent studies showing separation between flg22-dependent ROS production and MAPK activation using plants defective in gene expression of specific signaling components such as *MAPK*, *CDPKs* and *RbohD* [13], [19], [22], [78]. In agreement, we have recently shown that pretreatment with the chemical inhibitors Wm and TyrA23 impair flg22-induced ROS production but not MAPK phosphorylation [13]. Based upon the results of these chemical interference and genetic studies, it appears that MAPK pathway activation is not dependent upon flg22-stimulated changes in vesicular trafficking. Here, we have expanded on these studies showing that pre-treatment with Wm (Fig. 3), but not TyrA23 (S5 Fig.), disrupts DRP2B function to the extent that genetic loss of *DRP2B* no longer has a significant effect upon flg22-induced ROS production. As an inhibitor of PI-3 and PI-4 kinase activity, Wm impedes phosphorylation of phosphoinositol lipids within biological membranes, thus potentially disrupting the correct targeting of lipid-binding proteins to specific cellular membranes. For flg22-induced ROS production, Wm may affect the localization of one or more yet unidentified lipid-binding protein(s) in direct or indirect manners. DRP2B is a potential candidate as it contains a Pleckstrin-Homology (PH) domain, which is a lipid-binding domain that enables protein recruitment to phosphorylated phosphoinositides involved in vesicular trafficking or signal transduction; but the lipid binding properties of the DRP2B PH-domain have not been characterized. However, it is also likely that Wm affects (directly or indirectly) other components involved in flg22-induced ROS and/or endocytosis of FLS2. Wm-pretreatment results in strongly impaired ligand-induced endocytic degradation of FLS2 and a greater than 70% reduction of FLS2 accumulation in endosomes [9], [13], compared with the 20% reduction of FLS2 accumulation in endosomes in the *drpb2* mutant. Furthermore, flg22-elicitation resulted in increased ROS in *drp2b* but in decreased ROS after Wm-pretreatment (Fig. 3). Given the fact that Wm reduced ROS production in *drp2b* to the same level as in WT (Col-0), one explanation for this differential effect could be that besides impeding FLS2 endocytosis, Wm may have additional inhibitory effects on components required for ROS production that function upstream of DRP2B. We cannot exclude the possibility, however, that Wm affects components in separate pathway(s) than DRP2B.

Analysis of the *drp2b* mutant provided additional insights into how the diverse signaling branches may contribute to effective flg22-signaling and immunity against pathogenic bacteria. Our data supports a previously proposed model [20], [79] in which an important element of robust immunity is transcriptional reprogramming including *PR* gene expression at a relatively late phase of the immune response. Increased immune responses in the *RbohD*/Ca^2+^ branch (at least to the extent observed in *drp2b*) or normal induction of the MAPK branch were not sufficient to overcome decreased *PR1* gene induction or promote wildtype resistance to *Pto* DC3000 or *Pto hrcC^-^*. It is noteworthy that similar to *drp2b*, mutations in *SCD1*, a gene encoding a vesicular trafficking protein with putative Rab-GEF activity [80], [81], also result in a non-canonical combination of flg22-signaling responses [20]. These immune response defects are at least in part opposite to those described in this study for *drp2b* mutant plants. Reduced SCD1 protein levels lead to decreased flg22-induced ROS-production but increased *PR1* mRNA levels, as well as increased resistance to *Pto* DC3000. Thus, *SCD1* is a negative regulator [20] while *DRP2B* is a positive regulator (this study) of immunity against *Pto* DC3000. Similar to DRP2s, SCD1 is associated with clathrin-coated vesicles (CCVs) [80], [81]; but the exact role(s) of SCD1 in CCV formation or ligand-induced endocytosis of FLS2, or its functional relationship to DRP2B, remain unknown.

### Integration of DRP2B's role in immune signaling and trafficking

Overall, relatively little is known about a potential role(s) of flg22-induced endocytosis and subsequent degradation of FLS2 in modulating immune responses [13], [82]. A possible explanation for how loss of *DRP2B* may result in the non-canonical combination of immune defects may stem, at least in part, from DRP2B's function in ligand-induced endocytosis of FLS2. Drawing from mammalian studies, ligand-induced endocytosis of cell surface receptors may contribute to termination of signaling responses initiated at the PM [83], [84]. Internalization may also ensure contact between receptors and endosomal signaling components to initiate distinct signaling events from endosomes [85], [86].

As shown for mammalian Toll-like receptor 4 (TLR4), the PRR for the bacterial PAMP lipopolysaccharide (LPS), these functions are not mutually exclusive [87]. When disrupting cellular dynamin functions with Dynasore, a chemical inhibitor that generally blocks large GTPase activity including that of dynamins [88], or when expressing a dominant-negative dynamin protein (Dyn K44A), internalization of TLR4 and LPS are disrupted [89], [90]. Analogous to the non-canonical combination of immune defects in *drp2b* plants, inhibition of dynamin-dependent internalization results in increased signaling through the MyD88/NFκB-branch but decreased signaling through the TRAM-TRIFF-branch [89], [90]. Although much less is known about how trafficking of TLR5, the mammalian cell surface receptor for flagellin, affects immune signaling, flagellin-induced gene expression of pro-inflammatory cytokine and chemokines was significantly reduced after treatment Dynasore [91].

For the *Arabidopsis* FLS2-flg22 system, it is possible that the very early and transient signaling responses may be in part regulated through FLS2 receptor dynamics, such that delayed endocytosis may lead to heightened signaling from activated FLS2 at the PM. In support of this, *drp2b* null mutant plants display enhanced flg22-induced cytosolic Ca^2+^ levels and ROS production, which could be due to the 20% reduction of flg22-induced internalization of FLS2 (Fig. 7D). Endocytic internalization of FLS2 may contribute to a dampening of these early signaling responses. In *drp2b*, an increase in *PHI1* mRNA and callose deposition are likely a consequence of enhanced Ca^2+^ and ROS production, which are known to function upstream of these later responses. While FLS2 internalization into endosomes may affect some responses, others such as initiation of the MAPK-signaling branch may be independent of ligand-induced endocytosis of FLS2. A delay in FLS2 trafficking through the endosomal compartments could explain the reduced *PR1* mRNA levels in *drp2b*, and such correlation between flg22-induced FLS2 endocytosis and *PR1* mRNA expression may likely occur via indirect mechanisms due to the disconnect in timing (within 1 hour for FLS2 internalization and endocytic degradation versus 24 hours for induced *PR1* expression). Interestingly, the *Lycopersium esculentum* (tomato) receptor-like protein LeEix2 has been previously reported to require trafficking from the PM to endosomal compartments to initiate downstream signaling responses upon recognition of the fungal protein ethylene-inducing xylanase (EIX) [92]. Similar to our *drp2b* results, this study implicates dynamins in ligand-induced endocytosis based on the observation that treatment with the chemical inhibitor Dynasore leads to reduced EIX-induced endocytosis of transiently expressed LeEIX2-GFP in *Nicotiana benthamiana* [92]. In contrast to our study, however, Dynasore treatment results in reduction of all of the reported EIX-induced responses including impaired ROS production [92]. This difference in signaling response defects may reflect the use of Dynasore, which is known to affect activities of multiple DRPs and large GTPases [88], whereas our study has focused in the analysis of a loss-of-function mutant in a single gene (*DRP2B*). The number of DRPs in *N. benthamiana* has not been defined; but *A. thaliana* contains 16 different DRPs that fall into six subgroups and have diverse cellular functions and subcellular localizations [25].

In addition to FLS2, the activities of other PM-localized factors that function in flg22-responses may be regulated directly or indirectly by DRP2B. One potential candidate may be RbohD, as the diffusion and clustering of RbohD-GFP within the PM has been shown to be impaired in mutant plants lacking *Clathrin Heavy Chain 2 (CHC2)* [93], which similar to *DRP2B*, encodes a CCV component functioning in CME [94]. However, it remains to be determined whether mutant plants lacking *CHC2* display defects in RbohD-dependent ROS production [93]. Interestingly, immunoprecipitation experiments recently identified DRP2B and RbohD as two of 62 proteins that may form complex(es) with *Botrytis*-induced kinase 1 (BIK1) [58], a dual specificity receptor-like cytoplasmic kinase with roles in flg22-signaling and plant innate immunity via interactions with FLS2 and BAK1 [95]–[97]. BIK1 directly interacts with and phosphorylates RbohD on multiple serine residues in a calcium-independent manner [58], [72] that are critical for flg22-induced ROS production and contribute to immunity against *Pto* [18], [58], [72]. The functional significance of the potential (direct or indirect) interaction between DRP2B and BIK1, however, remains unknown. In the longer term, it will be interesting to determine whether the increased flg22-induced ROS production in *drp2b* may be due to defects in RbohD trafficking and whether these defects may be dependent on *BIK1*.

To date, very few vesicular trafficking proteins are implicated in affecting FLS2 endocytosis. As for DRP2B, consistent with being a CCV component with roles in CME [33], [34], DRP2B's role in ligand-induced endocytosis of FLS2 may occur via clathrin-dependent mechanisms; but we cannot exclude the possibility that DRP2B, like the mammalian dynamins [30], [31], may function in clathrin-independent endocytosis [35], [36]. Similar to DRP2B, the Arabidopsis ESCRT-1 subunit VPS37-1 was recently shown to be partially required for flg22-stimulated endocytosis of FLS2, in that *vps37-1* mutant plants exhibited an approximately 20% reduction in FLS2-GFP accumulation in endosomal compartments [14]. However unlike *drp2b*, *vsp37-1* plants do not display increased ROS production or callose deposition in response to flg22. One possible explanation is that VPS37-1 and DRP2B may regulate flg22-stimulated endocytosis of FLS2 at different trafficking steps, which could have differential effects on flg22-signaling. Consistent with this idea, VPS37-1 is predicted to function at the MVB/LE [14] while DRP2B is reported to localize and function at the PM [17], [18], [32], [33].

Flg22-induced polyubiquitination and subsequent degradation of FLS2 is dependent on two closely-related E3 ligases PUB12 and PUB13 [11]. Loss of *PUB12/13* results in elevated flg22-induced ROS and callose deposition [11]. While both *drp2b* and *pub12/13* mutants display increased RbohD-dependent responses, we did not observe a block in flg22-stimulated degradation of FLS2 in *drp2b* as reported for *pub12/13* [11]; but so far, the subcellular compartment(s) at which FLS2 accumulates in the *pub12/13* mutants remains to be determined. Furthermore in contrast to *drp2b*, mutations in *pub13* have been reported to result in increased accumulation of SA and *PR1* mRNA levels in the absence of any stimulus [98], which may contribute to the increased resistance to infection by various *Pto* strains in these *pub* mutants under certain growth conditions [11], [98]. Thus, it would seem that the increase in RbohD-dependent responses and flg22-dependent trafficking defects of FLS2 in *drp2b* and *pub12/13* plants may occur through different mechanisms.

In conclusion, we provide evidence that DRP2B, but not DRP2A, is required for robust ligand-induced endocytosis of FLS2. The identification of DRP2B as a novel component functioning in flg22-signaling and immunity against *Pto hrcC^−^* adds DRP2B to the relatively short list of vesicular trafficking proteins involved in PAMP-signaling and PTI. Moreover, our findings underline the importance of a functional vesicular trafficking network for plant innate immune responses for effective immunity against invading pathogenic microbes. In view of the described diversity in immune defects for only a limited number of mutants that affect FLS2 endocytic trafficking, a more refined set of genetic tools is needed to more distinctly dissect to what extent ligand-induced endocytosis of FLS2 contributes to the regulation of individual signaling pathways. As such, the *drp2b* mutant provides an intriguing tool to gain further insights into how vesicular trafficking and diverse flg22-signaling branches contribute to effective immunity against pathogenic bacteria.

## Supporting Information

S1 Fig ***drp2b*** **mutants display increased ROS in response to multiple PAMPs independent of** ***RbohD*** **and** ***FLS2*** **mRNA levels.** (A) Compared to Col-0 and *drp2a-1* (*2a-1*), peak ROS production (at 10–15 minutes post-elicitation) was significantly increased in *drp2b-2* (*2b-2*) after elicitation with 0.1 µM of active flg22 (black bars) (P<0.0001). Responses to inactive flg22*^A.tum^* (white bars) were not different between genotypes (P>0.5). Data were based on time-course experiment from Fig. 1C. (n = 24/genotype and treatment). (B) Compared to Col-0 (white bar) and *drp2a-1* (*2a-1*; gray bar), peak ROS production (10–15 minutes post elicitation) was significantly increased in *drp2b-2* (*2b-2*; black bar) after elicitation with 0.1 µM elf26 (P<0.005). (n = 32/genotype). (C) Using quantitative Real-Time PCR (qRT-PCR) with *At2g28390* as the reference gene, mRNA levels of *RbohD* were not significantly different between *drp2b-2* (*2b-2*; black bar) and Col-0 (white bar). Tissues were cut and prepared exactly as those for ROS experiments in 96-well plates and collected immediately prior to flg22-elicitation (n = 3/genotype; P = 0.9). (D) Based on experimental design and qRT-PCR as described in (C), mRNA levels of *FLS2* were not significantly different between *drp2b-2* (*2b-2*; black bar) and Col-0 (white bar) (P = 0.33). (n = 3/genotype). For (A - B), luminol-based ROS production is shown as Relative Light Units (RLU). For (A - D), all experiments were done in 4-5 week old leaf tissue and repeated more than three independent times with similar results. Values are mean ± SE. Different letters indicate significant differences while the same letter indicates no significant differences between samples based on Two tailed student's t-test. ROS experiments shown in the same panel were performed in the same 96-well plate at the same time to allow for direct comparison.
(PDF)

S2 Fig **Treatment with the calcium channel blocker LaCl_3_ abolishes flg22-induced increases in cytosolic Ca^2+^ levels and ROS production.** (A) After 0.1 µM flg22 elicitation, cytosolic Ca^2+^ levels were significantly elevated in leaf discs from 4–5 week old *drp2b-2* expressing the Ca^2+^-reporter Aequorin (*drp2b-2/AEQ*; closed symbols) compared to Col-0 expressing AEQ (Col-0/AEQ; open symbols). (n = 6/genotype and treatment). (B) LaCl_3_ abolished flg22-induced elevations of cytosolic Ca^2+^ levels to similar levels in *drp2b-2/*AEQ (closed symbols) compared to Col-0/AEQ (open symbols). 8-day old plants were pretreated with 10 mM LaCl_3_ (triangles) or water (squares) for 30 minutes, washed with water and then elicited with 0.1 µM flg22. (n = 6/genotype and treatment). (C) LaCl_3_ abolished flg22-induced ROS production to similar levels in *drp2b-2* (square) compared to Col-0 (diamond). Leaf discs from 4–5 week old plants were pretreated with 1 mM LaCl_3_ (open symbols) or water (closed symbols) for 30 minutes, washed with water and then elicited with 0.1 µM flg22. (n = 24/genotype and treatment). For all experiments, values are mean ± SE. Each experiment was repeated more than three independent times with similar results. Statistical analysis was done as in S1 Fig.
(PDF)

S3 Fig ***DRP2B*** **has no apparent role in the flg22-induced expression of** ***PER62, PER4 and NHL10*** **.** (A) and (B) Using qRT-PCR with *At2g28390* as the reference gene, mRNA levels of *PER62* and *PER4* were not significantly different between *drp2b-2* and Col-0 after elicitation for 30 minutes with water (white bars) or 1 µM flg22 (black bars). P-values for *PER62* or *PER4* mRNA levels between *drp2b-2* and Col-0 were all P>0.5. (n = 3/genotype and treatment). (C) Using qRT-PCR with *At2g28390* as the reference gene, mRNA levels of *NHL10* were not significantly different between *drp2b-2* and Col-0 after elicitation for 60 minutes with water (white bars) or 1 µM flg22 (black bars). P-values for *NHL10* mRNA levels between *drp2b-2* and Col-0 were all P>0.5. (n = 3/genotype and treatment). All experiments were done in leaf tissue of 4–5 week old plants and repeated at least 3 times with similar results. Values are mean ± SE. Statistical analysis was done as in S1 Fig.
(PDF)

S4 Fig **DRP2B has no apparent role in flg22-induced MAPK phosphorylation over 45 minutes post-elicitation.** (A) Quantification of protein bands from Fig. 2C using Bio-Rad Quantity One software. Data is presented as the ratio of either phosphorylated MPK6 (P-MPK6) or MPK3 (P-MPK3) relative to Calnexin protein levels. For quantification of P-MPK6 and P-MPK3, all data were normalized to the respective phosphorylated MAPK levels of the Col-0/10 minute timepoint. Quantified data represent the means ± SE from four independent biological repeats. (B) No apparent difference in flg22-induced phosphorylation of MPK3 and MPK6 was observed between Col-0 and *drp2b-2* over 45 minutes (min) after elicitation with 0.1 µM active flg22. Immunoblot analysis was done on total protein extracts probed with an antibody for phosphorylated MAPKs (P-MPK3 and P-MPK6). αCalnexin served as loading control. The depicted blot is representative of 3 individual experiments showing similar results. (C) Quantification of protein bands from S4B Fig. using Bio-Rad Quantity One software. Data is presented as the ratio of phosphorylated MPK6 (P-MPK6), MPK3 (P-MPK3), and an unknown MAPK (P-MPK?, potentially P-MPK4 or P-MPK11) relative to Calnexin protein expression. For quantification of P-MPK6, P-MPK3, and P-MPK?, all data was normalized to the respective phosphorylated MAPK levels of the Col-0/15 minute timepoint. Quantified data represent the means ± SE from four independent biological repeats. Statistical analysis was done as in S1 Fig.
(PDF)

S5 Fig **For** ***drp2b*** **, the increase in flg22-induced ROS production is not fully sensitive to Tyrphostin A23.** (A) After a one-hour pretreatment in the absence (square) or presence (triangle) of 100 µM Tyrphostin A23 (T23), ROS production was measured over 40 minutes in *drp2b-2* (closed symbols) and Col-0 (open symbols) in response to 1 µM flg22 (n = 24/treatment and genotype). (B) Total ROS production based on time-course experiments shown in (A) indicates that in *drp2b-2* (black bars), the increase in flg22-induced ROS production was not completely TyrA23-sensitive (*drp2b-2*+T23 vs Col-0+T23, P<0.025). Experiments were done using 4–5 week old leaf tissue and repeated at least 3 independent times with similar results. Values are mean ± SE. Statistical analysis was done as in S1 Fig. Relative Light Units, RLU.
(PDF)

S6 Fig **The Ca^2+^ channel blocker LaCl_3_ further reduces flg22-induced** ***PR1*** **mRNA levels in** ***drp2b-2*** **.** Using qRT-PCR with *At2g28390* as the reference gene, flg22-induced *PR1* mRNA levels were significantly reduced in *drp2b-2* (black bars) compared to Col-0 (white bars) 24 hr after co-treatment with flg22 and LaCl_3_ (P<0.05). Eight-day-old seedlings were treated with water, 10 mM LaCl_3_, 1 µM flg22 or co-treatment with 10 mM LaCl_3_ and 1 µM flg22 (flg22+ LaCl_3_). Water and LaCl_3_ treatment served as a controls. Data represent the mean ± SE of four or more independent experiments (n≥12). Statistical analysis was done as in S1 Fig.
(PDF)

S7 Fig **14-day old** ***drp2b-2*** **plants display similar phenotypic defects in response to flg22 or** ***Pto*** **DC3000 as described for 5-6 week old** ***drp2b-2*** **plants.** (A) No significant difference in the fresh weight of 14-day old plants was observed between *drp2b-2* (black bar) and *sid2-2* (gray bar) compared to Col-0 (white bar) (P>0.5). (n = 21/genotype). (B) In time-course experiments, ROS production was elevated in 14-day old *drp2b-2* plants (filled shapes) compared to Col-0 (open shapes) in response to 0.1 µM flg22. (n = 32/genotype). (C) In 14-day old plants, total ROS production was significantly increased in *drp2b-2* plants (black bar) compared to Col-0 (white bar) after elicitation with 0.1 µM flg22 (P<0.01). Data were based on time-course experiment shown in Figure (B). (D) Using qRT-PCR with *At2g28390* as the reference gene, *PR1 mRNA* levels were significantly reduced in *drp2b-2* (black bars) compared to Col-0 (white bars) at 24 hr after treatment with 1 µM flg22 (P<0.05). Treatment with water served as mock control. (n = 4/genotype and treatment). (E) Compared to Col-0 (white bars), bacterial growth was significantly increased in 14-day old *drp2b-2* plants (black bar) at 3 dpi with *Pto* DC3000lux (OD_600_ = 0.0005) as measured by serial dilution plating (P<0.05). *sid2-2* (gray bar) served as positive control (P<0.0001). (n = 8/genotype). (F) Using qRT-PCR, *PR1 mRNA* levels were significantly reduced in *drp2b-2* (black bars) compared to Col-0 (white bars) at 24 hr after treatment with *Pto* DC3000lux (OD_600_ = 0.0005) (P<0.009). Treatment with water (white bars) served as mock control. (n = 4/genotype). (G) Levels of SA in Col-0 (white bars) and *drp2b-2* (black bars) 12-day old plants treated for 24 hr with 1 µM flg22 +0.1% DMSO in water (P = 0.2; n = 3/genotype; each n contained 25 seedlings). Treatment with 0.1% DMSO in water served as mock control (P = 0.3; n = 2/genotype; each n contained 25 seedlings). Experiment was repeated twice with similar results. Unless stated differently, all experiments were done using 14-day old plants and repeated at least 3 independent times with similar results. Values are mean ± SE, and statistical analysis was done as in S1 Fig. ROS experiments shown in the same panel were performed in the same 96-well plate at the same time to allow for direct comparison. Relative Light Units, RLU.
(PDF)

S8 Fig **DRP2A is not required for robust flg22-induced endocytosis of FLS2.** (A) Col-0 *FLS2-GFP* and *drp2a-3 FLS2-GFP* homozygous F4 seedlings expressed similar levels of both endogenous FLS2 and FLS2-GFP as shown by immunoblot analyses of total protein extracts. αFLS2 detected both native FLS2 (open arrow) and FLS2-GFP (closed arrow), while αGFP detected FLS2-GFP only (closed arrow). αDRP2 was used to confirm *drp2a-3* mutants, and αMPK6 was used as a loading control. (B) In response to 1 µM flg22, no significant differences are observed in total ROS between Col-0 *FLS2-GFP* and *drp2a-3 FLS2-GFP* (P = 0.4) (n = 30 cotyledons/genotype). Relative Light Units, RLU. (C) Flg22-induced endocytosis of FLS2-GFP in Col-0 *FLS2-GFP* (Col-0) and *drp2a-3 FLS2-GFP* was determined by spinning disc confocal microscopy. Whole seedlings were treated with 1 µM flg22 to observe un-elicited (constitutive; 0 min) and ligand-induced (50–60 min) endocytosis of FLS2-GFP. Representative maximum-intensity projection images and zoomed insets of FLS2-GFP fluorescence are shown. Scale bars = 10 µm. (D) Quantification of FLS2-GFP in puncta at 0 min and 50–60 min after elicitation with 1 µM flg22 showed no significant differences between *drp2a-3* and Col-0 (0 min, P = 0.268; 50–60 min, P = 0.702). (n = 28–55 images analyzed per genotype/treatment). All experiments were done using 7-day old seedlings and were repeated at least three independent times with similar results. Values are mean ± SE, and statistical analysis was done as in S1 Fig.
(PDF)

S1 Table **List of oligonucleotide sequences used as primers.**
(PDF)

S1 File **Supplemental Materials and Methods.**
(PDF)
