# Supplementary material for: Loss of Arabidopsis thaliana Dynamin-Related Protein 2B Reveals Separation of Innate Immune Signaling Pathways
Source: PLoS Pathog. 2014 Dec 18;10(12):e1004578. doi: 10.1371/journal.ppat.1004578 (PMC4270792; doi:10.1371/journal.ppat.1004578)
Supplement: S1 Fig — drp2b mutants display increased ROS in response to multiple PAMPs independent of RbohD and FLS2 mRNA levels. (A) Compared to Col-0 and drp2a-1 (2a-1), peak ROS production (at 10–15 minutes post-elicitation) was significantly increased in drp2b-2 (2b-2) after elicitation with 0.1 µM of active flg22 (black bars) (P<0.0001). Responses to inactive flg22A.tum (white bars) were not different between genotypes (P>0.5). Data were based on time-course experiment from Fig. 1C. (n = 24/genotype and treatment). (B) Compared to Col-0 (white bar) and drp2a-1 (2a-1; gray bar), peak ROS production (10–15 minutes post elicitation) was significantly increased in drp2b-2 (2b-2; black bar) after elicitation with 0.1 µM elf26 (P<0.005). (n = 32/genotype). (C) Using quantitative Real-Time PCR (qRT-PCR) with At2g28390 as the reference gene, mRNA levels of RbohD were not significantly different between drp2b-2 (2b-2; black bar) and Col-0 (white bar). Tissues were cut and prepared exactly as those for ROS experiments in 96-well plates and collected immediately prior to flg22-elicitation (n = 3/genotype; P = 0.9). (D) Based on experimental design and qRT-PCR as described in (C), mRNA levels of FLS2 were not significantly different between drp2b-2 (2b-2; black bar) and Col-0 (white bar) (P = 0.33). (n = 3/genotype). For (A - B), luminol-based ROS production is shown as Relative Light Units (RLU). For (A - D), all experiments were done in 4-5 week old leaf tissue and repeated more than three independent times with similar results. Values are mean ± SE. Different letters indicate significant differences while the same letter indicates no significant differences between samples based on Two tailed student's t-test. ROS experiments shown in the same panel were performed in the same 96-well plate at the same time to allow for direct comparison. (PDF) [file ppat.1004578.s001.pdf]

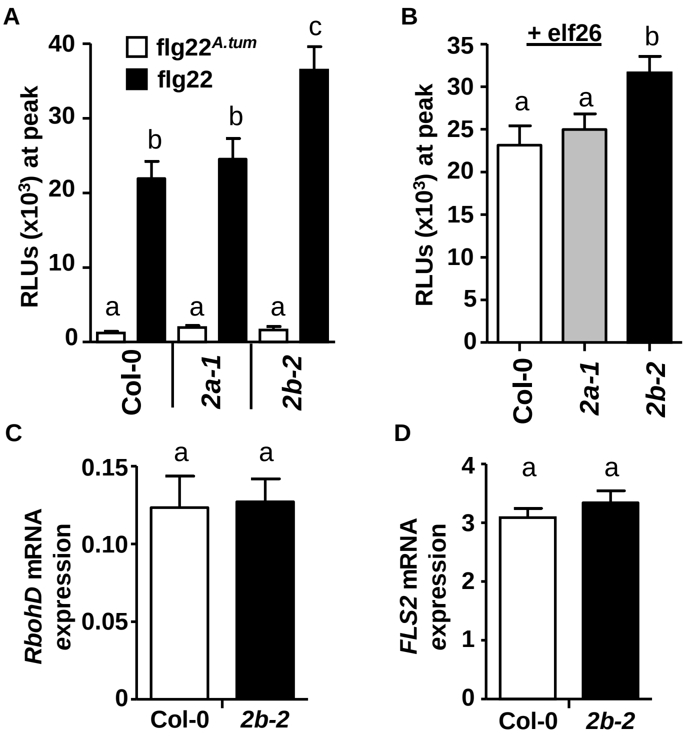

Figure S1. *drp2b* mutants display increased ROS in response to multiple PAMPs independent of *RbohD* and *FLS2* mRNA levels.

(A) Compared to Col-0 and *drp2a-1* (*2a-1*), peak ROS production (at 10-15 minutes post-elicitation) was significantly increased in *drp2b-2* (*2b-2*) after elicitation with 0.1µM of active flg22 (black bars) ($P<0.0001$). Responses to inactive flg22$^{A.tum}$ (white bars) were not different between genotypes ($P>0.5$). Data were based on time-course experiment from Figure 1C. (n=24/genotype and treatment). (B) Compared to Col-0 (white bar) and *drp2a-1* (*2a-1*; gray bar), peak ROS production (10-15 minutes post elicitation) was significantly increased in *drp2b-2* (*2b-2;* black bar) after elicitation with 0.1µM elf26 ($P<0.005$). (n=32/genotype). (C) Using quantitative Real-Time PCR (qRT-PCR) with *At2g28390* as the reference gene, mRNA levels of *RbohD* were not significantly different between *drp2b-2* (*2b-2;* black bar) and Col-0 (white bar). Tissues were cut and prepared exactly as those for ROS experiments in 96-well plates and collected immediately prior to flg22-elicitation (n=3/genotype; $P=0.9$). (D) Based on experimental design and qRT-PCR as described in (C), mRNA levels of *FLS2* were not significantly different between *drp2b-2* (*2b-2;* black bar) and Col-0 (white bar) ($P=0.33$). (n=3/genotype). For (A - B), luminol-based ROS production is shown as Relative Light Units (RLU). For (A - D), all experiments were done in 4-5 week old leaf tissue and repeated more than three independent times with similar results. Values are mean ± SE. Different letters indicate significant differences while the same letter indicates no significant differences between samples based on Two tailed student's t-test. ROS experiments shown in the same panel were performed in the same 96-well plate at the same time to allow for direct comparison.
