# Supplementary material for: Loss of Arabidopsis thaliana Dynamin-Related Protein 2B Reveals Separation of Innate Immune Signaling Pathways
Source: PLoS Pathog. 2014 Dec 18;10(12):e1004578. doi: 10.1371/journal.ppat.1004578 (PMC4270792; doi:10.1371/journal.ppat.1004578)
Supplement: S2 Fig — Treatment with the calcium channel blocker LaCl3 abolishes flg22-induced increases in cytosolic Ca2+ levels and ROS production. (A) After 0.1 µM flg22 elicitation, cytosolic Ca2+ levels were significantly elevated in leaf discs from 4–5 week old drp2b-2 expressing the Ca2+-reporter Aequorin (drp2b-2/AEQ; closed symbols) compared to Col-0 expressing AEQ (Col-0/AEQ; open symbols). (n = 6/genotype and treatment). (B) LaCl3 abolished flg22-induced elevations of cytosolic Ca2+ levels to similar levels in drp2b-2/AEQ (closed symbols) compared to Col-0/AEQ (open symbols). 8-day old plants were pretreated with 10 mM LaCl3 (triangles) or water (squares) for 30 minutes, washed with water and then elicited with 0.1 µM flg22. (n = 6/genotype and treatment). (C) LaCl3 abolished flg22-induced ROS production to similar levels in drp2b-2 (square) compared to Col-0 (diamond). Leaf discs from 4–5 week old plants were pretreated with 1 mM LaCl3 (open symbols) or water (closed symbols) for 30 minutes, washed with water and then elicited with 0.1 µM flg22. (n = 24/genotype and treatment). For all experiments, values are mean ± SE. Each experiment was repeated more than three independent times with similar results. Statistical analysis was done as in S1 Fig. (PDF) [file ppat.1004578.s002.pdf]

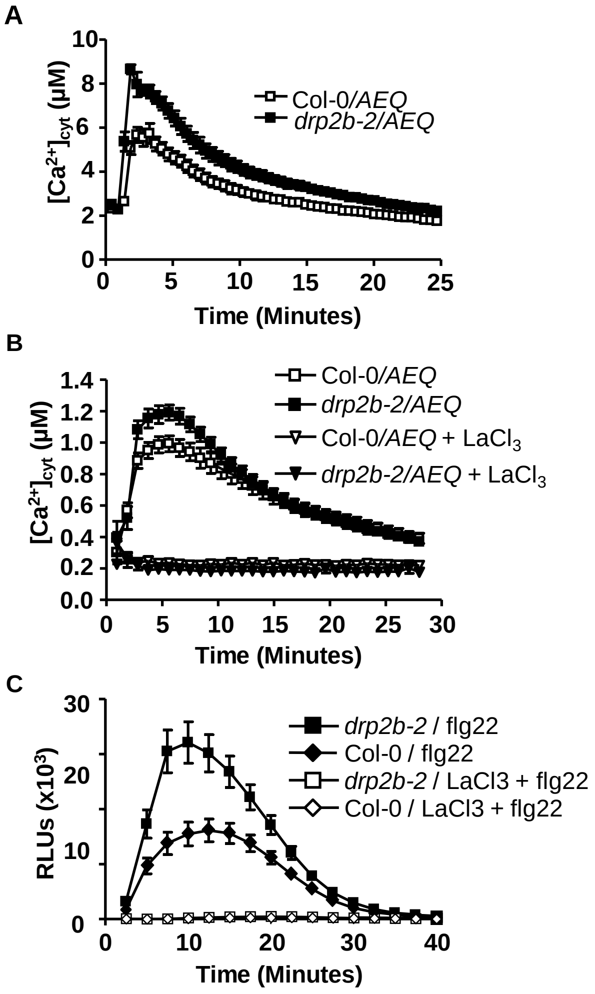

Figure S2. Treatment with the calcium channel blocker $LaCl_3$ abolishes flg22-induced increases in cytosolic Ca^{2+} levels and ROS production.

(A) After 0.1µM flg22 elicitation, cytosolic Ca^{2+} levels were significantly elevated in leaf discs from 4-5 week old *drp2b-2* expressing the Ca^{2+}-reporter Aequorin (*drp2b-2/AEQ*; closed symbols) compared to Col-0 expressing AEQ (Col-0/AEQ; open symbols). (n= 6/ genotype and treatment). (B) $LaCl_3$ abolished flg22-induced elevations of cytosolic Ca^{2+} levels to similar levels in *drp2b-2*/AEQ (closed symbols) compared to Col-0/AEQ (open symbols). 8-day old plants were pretreated with 10mM $LaCl_3$ (triangles) or water (squares) for 30 minutes, washed with water and then elicited with 0.1µM flg22. (n= 6/ genotype and treatment). (C) $LaCl_3$ abolished flg22-induced ROS production to similar levels in *drp2b-2* (square) compared to Col-0 (diamond). Leaf discs from 4-5 week old plants were pretreated with 1mM $LaCl_3$ (open symbols) or water (closed symbols) for 30 minutes, washed with water and then elicited with 0.1µM flg22. (n= 24/ genotype and treatment). For all experiments, values are mean ± SE. Each experiment was repeated more than three independent times with similar results. Statistical analysis was done as in Figure S1.
