# Supplementary material for: Loss of Arabidopsis thaliana Dynamin-Related Protein 2B Reveals Separation of Innate Immune Signaling Pathways
Source: PLoS Pathog. 2014 Dec 18;10(12):e1004578. doi: 10.1371/journal.ppat.1004578 (PMC4270792; doi:10.1371/journal.ppat.1004578)
Supplement: S3 Fig — DRP2B has no apparent role in the flg22-induced expression of PER62, PER4 and NHL10 . (A) and (B) Using qRT-PCR with At2g28390 as the reference gene, mRNA levels of PER62 and PER4 were not significantly different between drp2b-2 and Col-0 after elicitation for 30 minutes with water (white bars) or 1 µM flg22 (black bars). P-values for PER62 or PER4 mRNA levels between drp2b-2 and Col-0 were all P>0.5. (n = 3/genotype and treatment). (C) Using qRT-PCR with At2g28390 as the reference gene, mRNA levels of NHL10 were not significantly different between drp2b-2 and Col-0 after elicitation for 60 minutes with water (white bars) or 1 µM flg22 (black bars). P-values for NHL10 mRNA levels between drp2b-2 and Col-0 were all P>0.5. (n = 3/genotype and treatment). All experiments were done in leaf tissue of 4–5 week old plants and repeated at least 3 times with similar results. Values are mean ± SE. Statistical analysis was done as in S1 Fig. (PDF) [file ppat.1004578.s003.pdf]

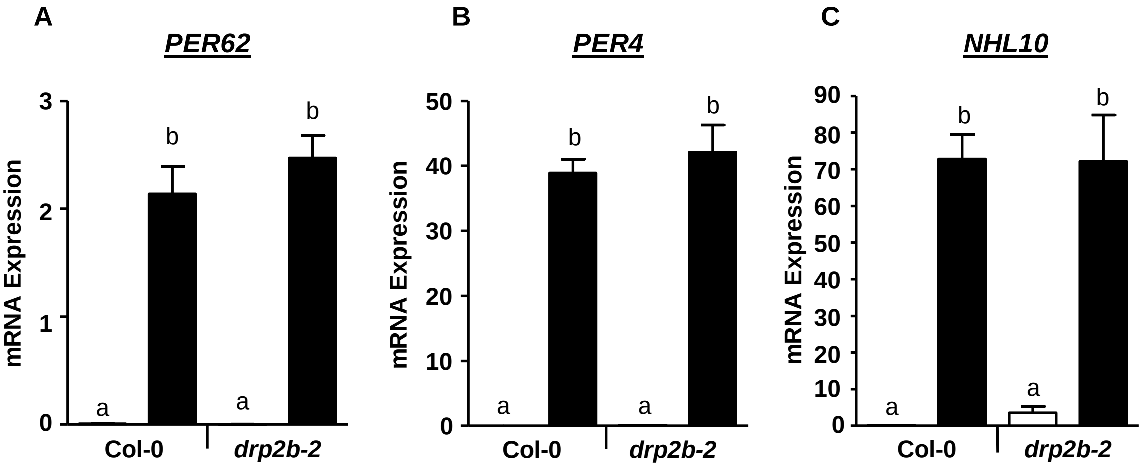

Figure S3. ***DRP2B*** **has no apparent role in the flg22-induced expression of** ***PER62, PER4 and NHL10.***

(A) and (B) Using qRT-PCR with *At2g28390* as the reference gene, mRNA levels of *PER62* and *PER4* were not significantly different between *drp2b-2* and Col-0 after elicitation for 30 minutes with water (white bars) or 1µM flg22 (black bars). P-values for *PER62* or *PER4* mRNA levels between *drp2b-2* and Col-0 were all P>0.5. (n=3/ genotype and treatment). (C) Using qRT-PCR with *At2g28390* as the reference gene, mRNA levels of *NHL10* were not significantly different between *drp2b-2* and Col-0 after elicitation for 60 minutes with water (white bars) or 1µM flg22 (black bars). P-values for *NHL10* mRNA levels between *drp2b-2* and Col-0 were all P>0.5. (n=3/ genotype and treatment). All experiments were done in leaf tissue of 4-5 week old plants and repeated at least 3 times with similar results. Values are mean ± SE. Statistical analysis was done as in Figure S1.
