# Supplementary material for: Loss of Arabidopsis thaliana Dynamin-Related Protein 2B Reveals Separation of Innate Immune Signaling Pathways
Source: PLoS Pathog. 2014 Dec 18;10(12):e1004578. doi: 10.1371/journal.ppat.1004578 (PMC4270792; doi:10.1371/journal.ppat.1004578)
Supplement: S4 Fig — DRP2B has no apparent role in flg22-induced MAPK phosphorylation over 45 minutes post-elicitation. (A) Quantification of protein bands from Fig. 2C using Bio-Rad Quantity One software. Data is presented as the ratio of either phosphorylated MPK6 (P-MPK6) or MPK3 (P-MPK3) relative to Calnexin protein levels. For quantification of P-MPK6 and P-MPK3, all data were normalized to the respective phosphorylated MAPK levels of the Col-0/10 minute timepoint. Quantified data represent the means ± SE from four independent biological repeats. (B) No apparent difference in flg22-induced phosphorylation of MPK3 and MPK6 was observed between Col-0 and drp2b-2 over 45 minutes (min) after elicitation with 0.1 µM active flg22. Immunoblot analysis was done on total protein extracts probed with an antibody for phosphorylated MAPKs (P-MPK3 and P-MPK6). αCalnexin served as loading control. The depicted blot is representative of 3 individual experiments showing similar results. (C) Quantification of protein bands from S4B Fig. using Bio-Rad Quantity One software. Data is presented as the ratio of phosphorylated MPK6 (P-MPK6), MPK3 (P-MPK3), and an unknown MAPK (P-MPK?, potentially P-MPK4 or P-MPK11) relative to Calnexin protein expression. For quantification of P-MPK6, P-MPK3, and P-MPK?, all data was normalized to the respective phosphorylated MAPK levels of the Col-0/15 minute timepoint. Quantified data represent the means ± SE from four independent biological repeats. Statistical analysis was done as in S1 Fig. (PDF) [file ppat.1004578.s004.pdf]

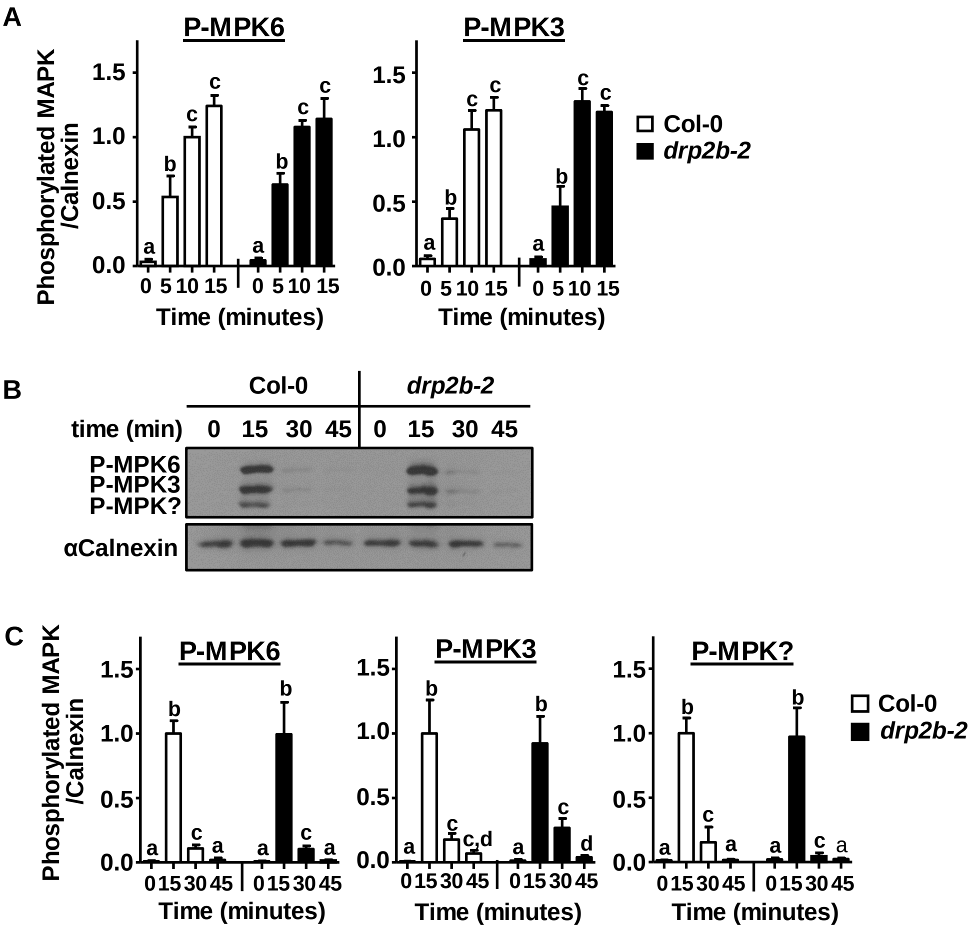

Figure S4. DRP2B has no apparent role in flg22-induced MAPK phosphorylation over 45 minutes post-elicitation.

(A) Quantification of protein bands from Figure 2C using Bio-Rad Quantity One software. Data is presented as the ratio of either phosphorylated MPK6 (P-MPK6) or MPK3 (P-MPK3) relative to Calnexin protein levels. For quantification of P-MPK6 and P-MPK3, all data were normalized to the respective phosphorylated MAPK levels of the Col-0 /10 minute timepoint. Quantified data represent the means ± SE from four independent biological repeats. (B) No apparent difference in flg22-induced phosphorylation of MPK3 and MPK6 was observed between Col-0 and *drp2b-2* over 45 minutes (min) after elicitation with 0.1µM active flg22. Immunoblot analysis was done on total protein extracts probed with an antibody for phosphorylated MAPKs (P-MPK3 and P-MPK6). αCalnexin served as loading control. The depicted blot is representative of 3 individual experiments showing similar results. (C) Quantification of protein bands from Figure S4B using Bio-Rad Quantity One software. Data is presented as the ratio of phosphorylated MPK6 (P-MPK6), MPK3 (P-MPK3), and an unknown MAPK (P-MPK?, potentially P-MPK4 or P-MPK11) relative to Calnexin protein expression. For quantification of P-MPK6, P-MPK3, and P-MPK?, all data was normalized to the respective phosphorylated MAPK levels of the Col-0/ 15 minute timepoint. Quantified data represent the means ± SE from four independent biological repeats. Statistical analysis was done as in Figure S1.
