# Supplementary material for: Loss of Arabidopsis thaliana Dynamin-Related Protein 2B Reveals Separation of Innate Immune Signaling Pathways
Source: PLoS Pathog. 2014 Dec 18;10(12):e1004578. doi: 10.1371/journal.ppat.1004578 (PMC4270792; doi:10.1371/journal.ppat.1004578)
Supplement: S5 Fig — For drp2b , the increase in flg22-induced ROS production is not fully sensitive to Tyrphostin A23. (A) After a one-hour pretreatment in the absence (square) or presence (triangle) of 100 µM Tyrphostin A23 (T23), ROS production was measured over 40 minutes in drp2b-2 (closed symbols) and Col-0 (open symbols) in response to 1 µM flg22 (n = 24/treatment and genotype). (B) Total ROS production based on time-course experiments shown in (A) indicates that in drp2b-2 (black bars), the increase in flg22-induced ROS production was not completely TyrA23-sensitive (drp2b-2+T23 vs Col-0+T23, P<0.025). Experiments were done using 4–5 week old leaf tissue and repeated at least 3 independent times with similar results. Values are mean ± SE. Statistical analysis was done as in S1 Fig. Relative Light Units, RLU. (PDF) [file ppat.1004578.s005.pdf]

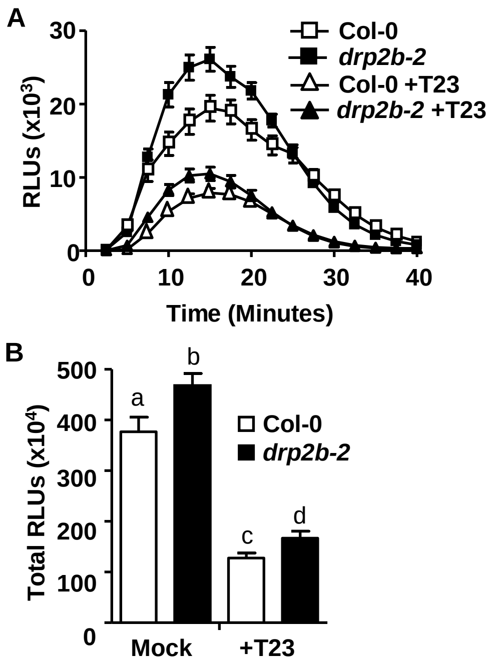

Figure S5. For *drp2b,* the increase in flg22-induced ROS production is not fully sensitive to Tyrphostin A23.

(A) After a one-hour pretreatment in the absence (square) or presence (triangle) of 100µM Tyrphostin A23 (T23), ROS production was measured over 40 minutes in *drp2b-2* (closed symbols) and Col-0 (open symbols) in response to 1µM flg22 (n=24/ treatment and genotype). (B) Total ROS production based on time-course experiments shown in (A) indicates that in *drp2b-2* (black bars), the increase in flg22-induced ROS production was not completely TyrA23-sensitive (*drp2b-2*+T23 vs Col-0+T23, $P<0.025$). Experiments were done using 4-5 week old leaf tissue and repeated at least 3 independent times with similar results. Values are mean ± SE. Statistical analysis was done as in Figure S1. Relative Light Units, RLU.
