# Supplementary material for: Loss of Arabidopsis thaliana Dynamin-Related Protein 2B Reveals Separation of Innate Immune Signaling Pathways
Source: PLoS Pathog. 2014 Dec 18;10(12):e1004578. doi: 10.1371/journal.ppat.1004578 (PMC4270792; doi:10.1371/journal.ppat.1004578)
Supplement: S6 Fig — The Ca2+ channel blocker LaCl3 further reduces flg22-induced PR1 mRNA levels in drp2b-2 . Using qRT-PCR with At2g28390 as the reference gene, flg22-induced PR1 mRNA levels were significantly reduced in drp2b-2 (black bars) compared to Col-0 (white bars) 24 hr after co-treatment with flg22 and LaCl3 (P<0.05). Eight-day-old seedlings were treated with water, 10 mM LaCl3, 1 µM flg22 or co-treatment with 10 mM LaCl3 and 1 µM flg22 (flg22+ LaCl3). Water and LaCl3 treatment served as a controls. Data represent the mean ± SE of four or more independent experiments (n≥12). Statistical analysis was done as in S1 Fig. (PDF) [file ppat.1004578.s006.pdf]

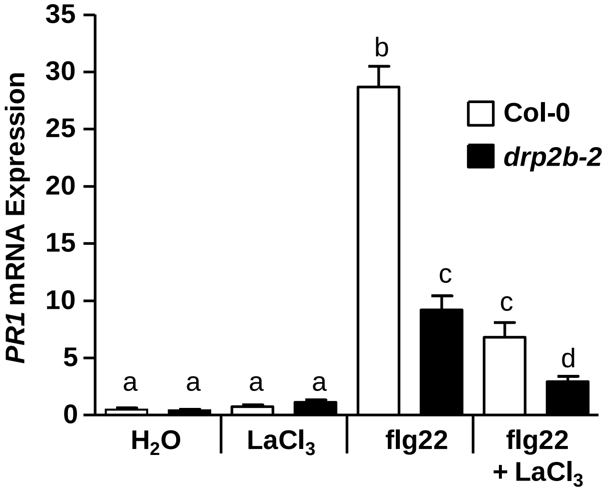

Figure S6. The Ca^{2+} channel blocker $LaCl_3$ further reduces flg22-induced *PR1* mRNA levels in *drp2b-2.*

Using qRT-PCR with *At2g28390* as the reference gene, flg22-induced *PR1* mRNA levels were significantly reduced in *drp2b-2* (black bars) compared to Col-0 (white bars) 24hr after co-treatment with flg22 and $LaCl_3$ ($P<0.05$). Eight-day-old seedlings were treated with water, 10 mM $LaCl_3$, 1 µM flg22 or co-treatment with 10 mM $LaCl_3$ and 1 µM flg22 (flg22+ $LaCl_3$). Water and $LaCl_3$ treatment served as a controls. Data represent the mean ± SE of four or more independent experiments ($n\geq12$). Statistical analysis was done as in Figure S1.
