# Supplementary material for: Loss of Arabidopsis thaliana Dynamin-Related Protein 2B Reveals Separation of Innate Immune Signaling Pathways
Source: PLoS Pathog. 2014 Dec 18;10(12):e1004578. doi: 10.1371/journal.ppat.1004578 (PMC4270792; doi:10.1371/journal.ppat.1004578)
Supplement: S7 Fig — 14-day old drp2b-2 plants display similar phenotypic defects in response to flg22 or Pto DC3000 as described for 5-6 week old drp2b-2 plants. (A) No significant difference in the fresh weight of 14-day old plants was observed between drp2b-2 (black bar) and sid2-2 (gray bar) compared to Col-0 (white bar) (P>0.5). (n = 21/genotype). (B) In time-course experiments, ROS production was elevated in 14-day old drp2b-2 plants (filled shapes) compared to Col-0 (open shapes) in response to 0.1 µM flg22. (n = 32/genotype). (C) In 14-day old plants, total ROS production was significantly increased in drp2b-2 plants (black bar) compared to Col-0 (white bar) after elicitation with 0.1 µM flg22 (P<0.01). Data were based on time-course experiment shown in Figure (B). (D) Using qRT-PCR with At2g28390 as the reference gene, PR1 mRNA levels were significantly reduced in drp2b-2 (black bars) compared to Col-0 (white bars) at 24 hr after treatment with 1 µM flg22 (P<0.05). Treatment with water served as mock control. (n = 4/genotype and treatment). (E) Compared to Col-0 (white bars), bacterial growth was significantly increased in 14-day old drp2b-2 plants (black bar) at 3 dpi with Pto DC3000lux (OD600 = 0.0005) as measured by serial dilution plating (P<0.05). sid2-2 (gray bar) served as positive control (P<0.0001). (n = 8/genotype). (F) Using qRT-PCR, PR1 mRNA levels were significantly reduced in drp2b-2 (black bars) compared to Col-0 (white bars) at 24 hr after treatment with Pto DC3000lux (OD600 = 0.0005) (P<0.009). Treatment with water (white bars) served as mock control. (n = 4/genotype). (G) Levels of SA in Col-0 (white bars) and drp2b-2 (black bars) 12-day old plants treated for 24 hr with 1 µM flg22 +0.1% DMSO in water (P = 0.2; n = 3/genotype; each n contained 25 seedlings). Treatment with 0.1% DMSO in water served as mock control (P = 0.3; n = 2/genotype; each n contained 25 seedlings). Experiment was repeated twice with similar results. Unless stated differently, all experim [file ppat.1004578.s007.pdf]

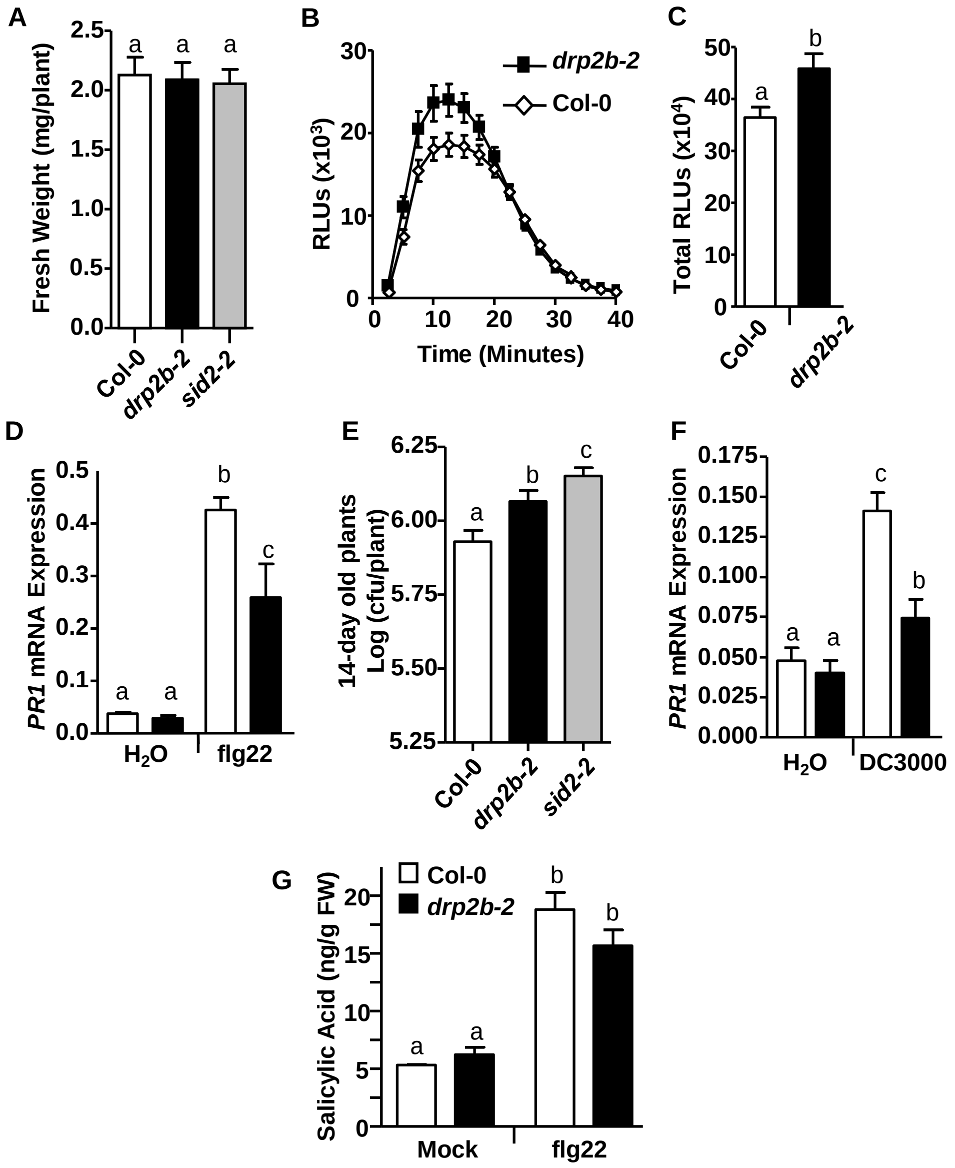

Figure S7. 14-day old *drp2b-2* plants display similar phenotypic defects in response to flg22 or *Pto* DC3000 as described for 5-6 week old *drp2b-2* plants.

(A) No significant difference in the fresh weight of 14-day old plants was observed between *drp2b-2* (black bar) and *sid2-2* (gray bar) compared to Col-0 (white bar) ($P>0.5$). (n=21/genotype). (B) In time-course experiments, ROS production was elevated in 14-day old *drp2b-2* plants (filled shapes) compared to Col-0 (open shapes) in response to 0.1µM flg22. (n=32/genotype). (C) In 14-day old plants, total ROS production was significantly increased in *drp2b-2* plants (black bar) compared to Col-0 (white bar) after elicitation with 0.1µM flg22 ($P<0.01$). Data were based on time-course experiment shown in Figure (B). (D) Using qRT-PCR with *At2g28390* as the reference gene, *PR1 mRNA* levels were significantly reduced in *drp2b-2* (black bars) compared to Col-0 (white bars) at 24hr after treatment with 1µM flg22 ($P<0.05$). Treatment with water served as mock control. (n=4/genotype and treatment). (E) Compared to Col-0 (white bars), bacterial growth was significantly increased in 14-day old *drp2b-2* plants (black bar) at 3 dpi with *Pto* DC3000lux (OD_{600}=0.0005) as measured by serial dilution plating ($P<0.05$). *sid2-2* (gray bar) served as positive control ($P<0.0001$). (n=8/genotype). (F) Using qRT-PCR, *PR1 mRNA* levels were significantly reduced in *drp2b-2* (black bars) compared to Col-0 (white bars) at 24 hr after treatment with *Pto* DC3000lux (OD_{600}=0.0005) ($P<0.009$). Treatment with water (white bars) served as mock control.

Figure S7 continued.

(n=4/genotype). (G) Levels of SA in Col-0 (white bars) and *drp2b-2* (black bars) 12-day old plants treated for 24 hr with 1 µM flg22 + 0.1% DMSO in water (P=0.2; n=3/genotype; each n contained 25 seedlings). Treatment with 0.1% DMSO in water served as mock control (P=0.3; n=2/genotype; each n contained 25 seedlings). Experiment was repeated twice with similar results. Unless stated differently, all experiments were done using 14-day old plants and repeated at least 3 independent times with similar results. Values are mean ± SE, and statistical analysis was done as in Figure S1. ROS experiments shown in the same panel were performed in the same 96-well plate at the same time to allow for direct comparison. Relative Light Units, RLU.
