# Supplementary material for: Loss of Arabidopsis thaliana Dynamin-Related Protein 2B Reveals Separation of Innate Immune Signaling Pathways
Source: PLoS Pathog. 2014 Dec 18;10(12):e1004578. doi: 10.1371/journal.ppat.1004578 (PMC4270792; doi:10.1371/journal.ppat.1004578)
Supplement: S8 Fig — DRP2A is not required for robust flg22-induced endocytosis of FLS2. (A) Col-0 FLS2-GFP and drp2a-3 FLS2-GFP homozygous F4 seedlings expressed similar levels of both endogenous FLS2 and FLS2-GFP as shown by immunoblot analyses of total protein extracts. αFLS2 detected both native FLS2 (open arrow) and FLS2-GFP (closed arrow), while αGFP detected FLS2-GFP only (closed arrow). αDRP2 was used to confirm drp2a-3 mutants, and αMPK6 was used as a loading control. (B) In response to 1 µM flg22, no significant differences are observed in total ROS between Col-0 FLS2-GFP and drp2a-3 FLS2-GFP (P = 0.4) (n = 30 cotyledons/genotype). Relative Light Units, RLU. (C) Flg22-induced endocytosis of FLS2-GFP in Col-0 FLS2-GFP (Col-0) and drp2a-3 FLS2-GFP was determined by spinning disc confocal microscopy. Whole seedlings were treated with 1 µM flg22 to observe un-elicited (constitutive; 0 min) and ligand-induced (50–60 min) endocytosis of FLS2-GFP. Representative maximum-intensity projection images and zoomed insets of FLS2-GFP fluorescence are shown. Scale bars = 10 µm. (D) Quantification of FLS2-GFP in puncta at 0 min and 50–60 min after elicitation with 1 µM flg22 showed no significant differences between drp2a-3 and Col-0 (0 min, P = 0.268; 50–60 min, P = 0.702). (n = 28–55 images analyzed per genotype/treatment). All experiments were done using 7-day old seedlings and were repeated at least three independent times with similar results. Values are mean ± SE, and statistical analysis was done as in S1 Fig. (PDF) [file ppat.1004578.s008.pdf]

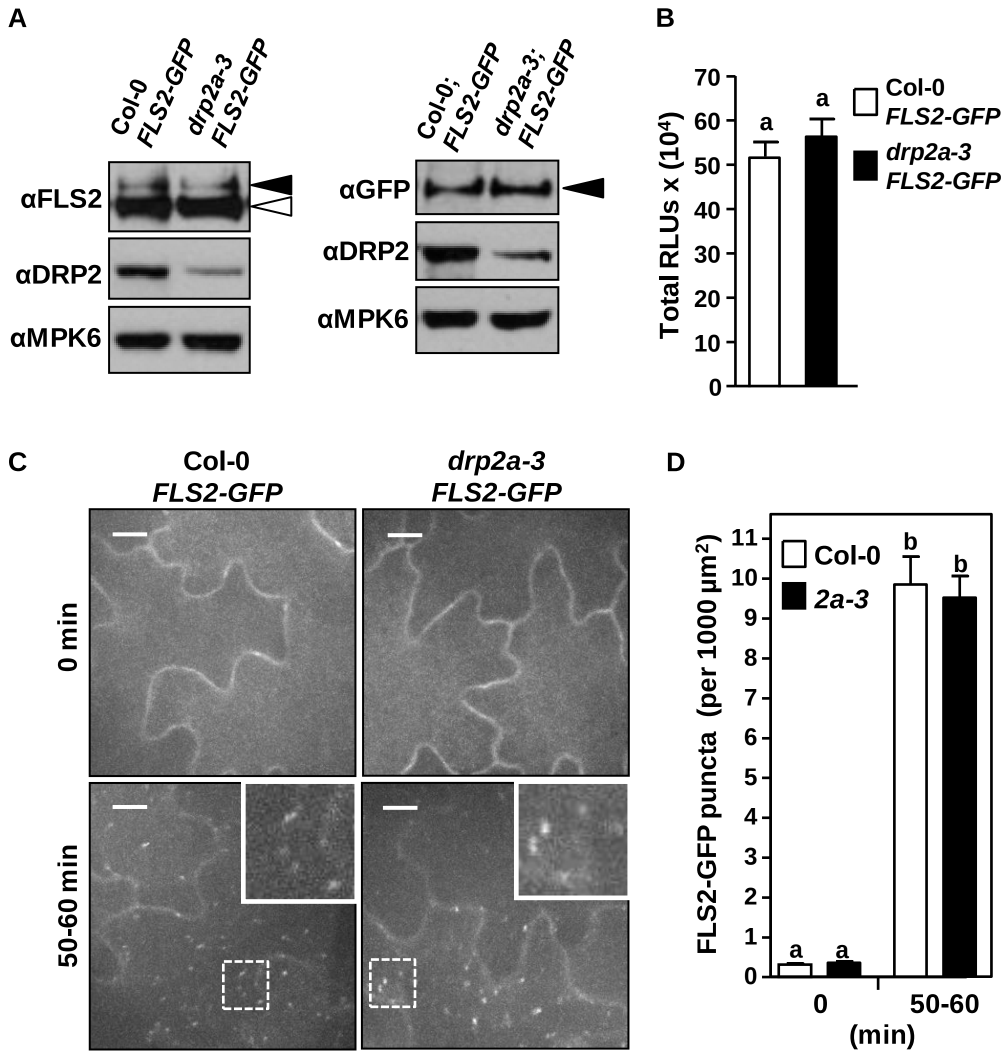

Figure S8. DRP2A is not required for robust flg22-induced endocytosis of FLS2.
(A) Col-0 *FLS2-GFP* and *drp2a-3 FLS2-GFP* homozygous F4 seedlings expressed similar levels of both endogenous FLS2 and FLS2-GFP as shown by immunoblot analyses of total protein extracts. αFLS2 detected both native FLS2 (open arrow) and FLS2-GFP (closed arrow), while αGFP detected FLS2-GFP only (closed arrow). αDRP2 was used to confirm *drp2a-3* mutants, and αMPK6 was used as a loading control. (B) In response to 1 µM flg22, no significant differences are observed in total ROS between Col-0 *FLS2-GFP* and *drp2a-3 FLS2-GFP* (P=0.4) (n=30 cotyledons/genotype). Relative Light Units, RLU. (C) Flg22-induced endocytosis of FLS2-GFP in Col-0 *FLS2-GFP* (Col-0) and *drp2a-3 FLS2-GFP* was determined by spinning disc confocal microscopy. Whole seedlings were treated with 1 µM flg22 to observe un-elicited (constitutive; 0 min) and ligand-induced (50-60 min) endocytosis of FLS2-GFP. Representative maximum-intensity projection images and zoomed insets of FLS2-GFP fluorescence are shown. Scale bars=10 µm. (D) Quantification of FLS2-GFP in puncta at 0 min and 50-60 min after elicitation with 1 µM flg22 showed no significant differences between *drp2a-3* and Col-0 (0 min, P=0.268; 50-60 min, P=0.702). (n=28-55 images analyzed per genotype/treatment). All experiments were done using 7-day old seedlings and were repeated at least three independent times with similar results. Values are mean ± SE, and statistical analysis was done as in Figure S1.
