# Supplementary material for: Loss of Arabidopsis thaliana Dynamin-Related Protein 2B Reveals Separation of Innate Immune Signaling Pathways
Source: PLoS Pathog. 2014 Dec 18;10(12):e1004578. doi: 10.1371/journal.ppat.1004578 (PMC4270792; doi:10.1371/journal.ppat.1004578)
Supplement: S1 Table — List of oligonucleotide sequences used as primers. (PDF) [file ppat.1004578.s009.pdf]

Purpose	Primer Name	Sequence (5'-3')
genotyping	drp2b-2 F	ATAGCCTAATTGGGCATCCAG
genotyping	drp2b-2 R	TATAGCATCGTTGTGCTGTGC
genotyping	drp2a-1 F	GGTTGATAACCATCTGCCTCC
genotyping	drp2a-1 R	AAACAACTGACATTCCATGGG
genotyping	drp2b-5 F	TTGCTATGTGTTTGTGATTGAAAC
genotyping	drp2b-5 R	GACAAAGGCATCAAGCAGTTC
genotyping	drp2a-3 F	CCTCCTTTCAAGCGAGAGG
genotyping	drp2a-3 R	TGCTTTTTGGTATAGCCAAGC
genotyping	Apoaequorin F	ATACCAATCCAACCCTAATCCTAAAATAC
genotyping	Apoaequorin R	TATTATAAGAAGGTTCCTCTTTCCTCCTA
genotyping	AtrbohD F	ATGAAAATGAGACGAGGCAATTC
genotyping	AtrbohD R	GGATACTGATCATAGGCGTGGCTCCA
genotyping	LBb1	GCGTGGACCGCTTGCTGCAACT
genotyping	FLS2-3xMyc-EGFP F	AAACTTGGAGGACACCATTCAC
genotyping	FLS2-3xMyc-EGFP R	TGTTCACCGTTCAAGTCTTCCTC
genotyping	sid2-2 F1	TTCTTCATGCAGGGGAGGAG
genotyping	sid2-2 F2	CAACCACCTGGTGCACCAGC
genotyping	sid2-2 R	AAGCAAAATGTTTGAGTCAGCA
qRT-PCR	AtrbohD F	TCGGTGACTGGACACGTAAGC
qRT-PCR	AtrbohD R	GTTGCAACGACTGAAGCATGG
qRT-PCR	FLS2 F	TCTGATGAAACTTAGAGGCAAAGCG
qRT-PCR	FLS2 R	CGTAACAGAGTTTGGCAAAGTCG
qRT-PCR	FRK1 F	ATCTTCGCTTGGAGCTTCTC
qRT-PCR	FRK1 R	TGCAGCGCAAGGACTAGAG
qRT-PCR	PHI1 F	TTGGTTTAGACGGGATGGTG
qRT-PCR	PHI1 R	ACTCCAGTACAAGCCGATCC
qRT-PCR	PR1 F	GCAATGGAGTTTGTGGTCAC
qRT-PCR	PR1 R	GTTCACATAATTCCCACGAGG
qRT-PCR	At2g28390 F	AACTCTATGCAGCATTTGATCCACT
qRT-PCR	At2g28390 R	TGATTGCATATCTTTATCGCCATC
qRT-PCR	WRKY33 F	AGCAAAGAGATGGAAAGGGGACAA
qRT-PCR	WRKY33 R	GCACTACGATTCTCGGCTCTCTCA
qRT-PCR	WRKY40 F	TGCGAGTTGAAGAAGATCCACCGA
qRT-PCR	WRKY40 R	TCCGAGAGCTTCTTGTTCTCAGCA
qRT-PCR	PER62 F	GCGATCTCGTCACTCTTGTTG
qRT-PCR	PER62 R	CGTGATAAACCCACATGCAG
qRT-PCR	PER4 F	GCGATCTCGTCACTCTTGTTG
qRT-PCR	PER4 R	CGTGATAAACCCACATGCAG
qRT-PCR	NHL10 R	TTCCTGTCCGTAACCCAAAC
qRT-PCR	NHL10	CCCTCGTAGTAGGCATGAGC

Table S1 List of oligonucleotide sequences used as primers.
