# Supplementary material for: Loss of Arabidopsis thaliana Dynamin-Related Protein 2B Reveals Separation of Innate Immune Signaling Pathways
Source: PLoS Pathog. 2014 Dec 18;10(12):e1004578. doi: 10.1371/journal.ppat.1004578 (PMC4270792; doi:10.1371/journal.ppat.1004578)
Supplement: S1 File — Supplemental Materials and Methods. (PDF) [file ppat.1004578.s010.pdf]

File S1. Supporting Materials and Methods

Cytosolic Calcium Measurements

Transient increases in cytosolic Ca^{2+} were monitored in leaf discs ($1.1 cm^2$, cut into halves) or cotyledons of eight-day old plants as described in Materials and Methods. For $LaCl_3$ treatments, plant tissue was pre-treated by floating cotyledons of eight-day old plants in water-containing 10 mM Lanthanum (III) chloride [40] ($LaCl_{3;}$ Santa Cruz Biotechnology; Dallas, TX) for thirty minutes. Before elicitation, the solution was removed and 0.1 µM flg22 was added to the wells. Luminescence was acquired using a Glomax 96 microplate Luminometer (Promega) scanning each row in five-second intervals. Calcium concentration was calculated as described [41].

Apoplastic ROS Production after $LaCl_3$ or TyrA23 pretreatment

Luminol-based ROS production in leaf tissue was performed as described [36] using indicated PAMP concentrations. For inhibitor treatment, leaf tissue was pre-treated by floating leaf discs in water-containing 1 mM $LaCl_3$ for thirty minutes prior to subsequent flg22-elicitation. For inhibitor treatments, leaf tissue was pre-treated by floating leaf discs in water-containing 100 µM Tyrphostin A23 (TyrA23; Sigma-Aldrich; St. Louis, MO) for one hour prior to subsequent flg22-elicitation [13]. All ROS experiments shown in a same panel were performed in the same 96-well plate at the same time to allow for direct comparison.

Quantitative Real-Time PCR (qRT-PCR) analysis

For *PER62, PER4 and* NHL10, three leaves of five-to-six week old plants were syringe infiltrated with flg22 allowed to dry and then placed at 22˚C. Tissue was flash-frozen in liquid nitrogen at indicated times. For all *PR1* mRNA expression experiments in the presence of absence of LaCl3, four eight-day old plants were transferred to eppendorf tubes containing 1 mL of dH_2O and incubated for 15–20 h prior to treatment. For $LaCl_3$ treatments, plant tissue was floated in water containing 10 mM $LaCl_3$ with or without 0.1 µM flg22 for 24 hours, after which plant tissue was flash-frozen in liquid nitrogen. Total RNA was isolated from collected tissue using Trizol Reagent (Sigma) according to the manufacturer's protocol and processed for qRT-PCR as described previously [52,78] using gene-specific primers (Table S1) and *At2g28390* as a reference gene.

Quantification of P-MPK6 and P-MPK3

Scanned images of protein bands detected on X-ray film were quantified using Quantity One software (Bio-Rad, Hercules, CA). Protein band intensities (P-MPK6, P-MPK3 and the loading control Calnexin) were each normalized to background. Data was plotted as the combined normalized intensities of P-MPK6 and P-MPK3 divided by the normalized intensities of Calnexin for each sample. Quantified data represents the means ± SE from 4 independent biological repeats.

Hormone analysis

Plants were grown on 1/2 strength MS plates + 1% sucrose under long day conditions (16 hours light/8 hours dark) under which *drp2b-2* plants showed increased susceptibility to *Pto* strains compared to wild-type (data not shown). For each sample analyzed, twenty-five 10-day old plants were transferred to one well of a 6-well sterile tissue culture dish with 7.5 mL of sterile dH_2O. After 16-20 hours, water was exchanged for 5 mL of 1uM flg22 in water + 0.1% DMSO. Incubation in water + 0.1% DMSO alone served as a mock-treatment. After 24 hours of flg22 or mock-treatment, excess water was gently removed and all 25 seedlings for each sample were placed in a 2 mL collection tube, which was weighed and flash-frozen in liquid nitrogen.

Frozen tissue samples were sent to the Proteomics & Mass Spectrometry Facility at the Danforth Plant Science Center (St. Louis, MO, USA; http://www.danforthcenter.org/scientists-research/core-technologies/proteomics-mass-spectrometry/services/targeted-metabolomics) for hormone analysis by liquid chromatography–tandem mass spectroscopy (LC-MS/MS) based on Mutka et al. (2013) and Chen et al (2009). Frozen tissue samples were ground with the TissueLyzer II (Qiagen), at 20 Hz/sec for 30 sec intervals until tissue was a fine powder. To each sample, 900 µL of ice cold MeOH/ACN (1:1 v/v) and 10 µL of a 2.5 µM deuterium-labeled SA standard (D5SA; ICON Isotopes, NJ, USA) was added, and the samples were homogenized with the TissueLyser II for 2 minutes at frequency of 15 Hz/sec, then centrifuged at 16,000 g for 5 minutes at 4^oC. The supernatants were transferred to new 2 mL tubes and the pellets were re-extracted as previously described. The second supernatant was combined to the first one and dried down. The dried pellets were dissolved in 200 µL of 30% methanol, then centrifuged again to remove un-dissolved material and the supernatant was transferred to vials for LC-MS/MS analysis. The injected volume of the sample was 50 µL.

The LC–MS/MS system used for SA analysis is composed of a Shimadzu LC system with two Shimadzu solvent delivery pumps (model LC10AD), a Shimadzu integrated controller (SCL10Avp), a Valco two-position diverter valve, and a LEAP CTC PAL autosampler with a 50 µL sample loop. This LC system is interfaced with an AB Sciex 4000 QTRAP mass spectrometer equipped with a TurboIonSpray (TIS) electrospray ion source. Source parameters were set as follows: curtain gas, 25 arbitrary units (a.u.); source gas 1, 50 a.u.; source gas 2, 50 a.u.; collision activated dissociation, high; interface heater, on; temperature, 550 ºC; ionspray voltage, –4500. Both quadruples (Q1 and Q3) were set to unit resolution. Analyst software (version 1.5) was used to control sample acquisition and data analysis. The 4000 QTRAP mass spectrometer was tuned and calibrated according to the manufacturer's recommendations. SA was detected using MRM transitions that were previously optimized using a standard and a deuterium-labeled standard.

For LC separation, a monolithic C18 column (Onyx, 4.6 mm x 100 mm, Phenomenex) with a guard cartridge was used flowing at 1 $mL.min^{-1}$. The gradient was from 40% solvent A (0.1% [v/v] acetic acid in Milli-Q water), held for 2 min, to 100% solvent B (90% acetonitrile [v/v] with 0.1% acetic acid [v/v] in 5 min. The LC was then ramped back to initial conditions (40% solvent A) in 1 min and re-equilibrated for an additional 2 min. For quantification, a series of standard samples containing different concentrations of SA was prepared. The peak area in samples was first normalized in the same way as used for the standard samples and then quantified according to the standard curve.

References

Mutka AM, Fawley S, Tsao T, Kunkel BN. (2013) Auxin promotes susceptibility to *Pseudomonas syringae* via a mechanism independent of suppression of salicylic acid-mediated defenses. *Plant J* 74(5): 746-54.

Chen Q, Zhang B, Hicks LM, Wang S, Jez JM. (2009) A liquid chromatography-tandem mass spectrometry-based assay for indole-3-acetic acid-amido synthetase. *Anal Biochem* 390(2): 149-54.
